# Design and Synthesis of N-Doped Carbons as Efficient Metal-Free Catalysts in the Hydrogenation of 1-Chloro-4-Nitrobenzene

**DOI:** 10.3390/ijms25052515

**Published:** 2024-02-21

**Authors:** Juan-José Villora-Picó, Antonio Sepúlveda-Escribano, María-Mercedes Pastor-Blas

**Affiliations:** Laboratory of Advanced Materials, Department of Inorganic Chemistry—University Institute of Materials of Alicante, University of Alicante, P.O. Box 99, E-03080 Alicante, Spain; jj.villora@ua.es (J.-J.V.-P.); asepul@ua.es (A.S.-E.)

**Keywords:** N-doped carbon, metal-free catalysts, hydrogenation of 1-chloro-4-nitrobenzene, melamine, citrate

## Abstract

Metal-free catalysts based on nitrogen-doped porous carbons were designed and synthesized from mixtures of melamine as nitrogen and carbon sources and calcium citrate as carbon source and porogen system. Considering the physicochemical and textural properties of the prepared carbons, a melamine/citrate ratio of 2:1 was selected to study the effect of the pyrolysis temperature. It was observed that a minimum pyrolysis temperature of 750 °C is required to obtain a carbonaceous structure. However, although there is a decrease in the nitrogen amount at higher pyrolysis temperatures, a gradual development of the porosity is produced from 750 °C to 850 °C. Above that temperature, a deterioration of the carbon porous structure is produced. All the prepared carbon materials, with no need for a further activation treatment, were active in the hydrogenation reaction of 1-chloro-4-nitrobenzene. A full degree of conversion was reached with the most active catalysts obtained from 2:1 melamine/citrate mixtures pyrolyzed at 850 °C and 900 °C, which exhibited a suitable compromise between the N-doping level and developed mesoporosity that facilitates the access of the reactants to the catalytic sites. What is more, all the materials showed 100% selectivity for the hydrogenation of the nitro group to form the corresponding chloro-aniline.

## 1. Introduction

Functionalized aromatic amines are intermediates in the synthesis of a wide variety of compounds with applications in the chemical industry such as dyes, drugs, herbicides and pesticides [1]. They can be easily produced by the reduction of aromatic nitro compounds [2], which, in turn, are toxic and harmful to human health and are frequently found in industrial effluents [3]. Developing green methodologies to transform toxic compounds into benign value-added products is the goal of relevant many research efforts nowadays [4]. The catalytic reduction of nitroarenes to produce the corresponding aniline is a crucial reaction in the chemical industry. However, the control of chemoselectivity when other reducible groups are present in the same nitroarene molecule is challenging.

The traditional Béchamp process of reduction of nitroarenes, developed in 1854 [5], involved the use of iron as a reductant. Other reductants such as tin and zinc in acid media and sulfides such as NaSH or H_2_S [6] were used as well. This process produced huge quantities of metallic waste, so new technologies for the reduction of nitroarenes, which used mild reductants such as formic acid [7,8], sodium borohydride [9,10] or hydrazine hydrate [11], were developed. Nevertheless, these are not exempt from some issues associated with the secure handling of formic acid or the formation of byproducts such as sodium borohydride. In this work, the use of benign molecular hydrogen has been preferred for environmental reasons as it is one of the cleanest reductants. In addition, hydrogen is non-toxic and much lighter than air, which permits a relatively rapid dissipation in case of leakage [12,13].

Reduction of nitroarenes requires the use of a catalyst, which can be either homogeneous or heterogeneous. The use of homogeneous catalysts based on metal complexes containing Fe [14], Mo [15] or Au [16] has been reported. Nevertheless, heterogeneous catalysts are preferred due to their easy separation and recyclability. They are usually based on noble metals (Pt [17,18], Pd [19,20], Au [21], Ru [22], Ir [23]) supported on a variety of materials exhibiting considerably high surface area. It has been observed that the strength of the interaction between the support and the noble metal catalyst produces good conversions; however, there are still issues associated with selectivity.

Although the activation of the nitro group is relatively easy during the hydrogenation reaction on metal-supported catalysts, the catalytic selectivity is usually low due to the production of intermediate species and competitive hydrogenation of other potentially reducible groups such as cyano, alkenyl, carbonyl and halide groups included in the same nitroarene molecule [24,25].

Taking into account the high price but low availability and selectivity of noble metals [14,15], alternatives based on non-noble metals (catalysts based on cobalt [12], nickel [26] iron [27] or copper [28]) were investigated. However, metal sintering and catalyst deactivation due to leaching of the metallic active phase to the reaction medium are still unsolved issues. Currently, the use of metal-free catalysts is gaining interest, for the sake of overcoming the problems inherent to metal-based catalysts [29].

The high interest of the chemical industry in the hydrogenation of nitroarenes to selectively produce anilines directed our research efforts towards the synthesis of green, efficient, economical and stable metal-free catalysts with high catalytic performance and total selectivity. Carbon materials are good candidates due to due to their high stability, low price and easy preparation [30,31]. Activated carbon has widely been used as support for metal catalysts and can also serve as a catalyst without the need for a metallic phase. However non-functionalized carbon is not a suitable catalyst due to the uniform charge distribution in the carbon lattice, which weakens the interaction between the reactants (H_2_ and the nitro group in the particular case of the hydrogenation of nitroarenes) and the carbon surface. Heteroatom doping has developed a great interest because it is capable of changing the electronic structure of carbon and also introduces defects in the carbon matrix that can act as potential active catalytic sites. N doping has become very popular as the N atom, with an atomic radius (0.65 Å) similar to that of the C atom (0.70 Å) can be easily inserted into the graphene lattice plane of the sp_2_ carbon structure. In addition, the larger electronegativity of N (3.04) compared to that of C (2.55), produces a charge redistribution on the C atoms adjacent to N, which favors the adsorption and activation of hydrogen molecules and nitro groups [32,33]. N can be inserted in the carbon lattice in the form of pyridinic, pyrrolic and quaternary nitrogen and pyridine-N-oxide species [34]. These nitrogen species play a crucial role in the catalytic process as they modify the carbon electron transport properties differently. The change in the arrangement of electron clouds in the carbon matrix results in the creation of unpaired electrons and incomplete saturated valences which affect carbon adsorption properties, mainly for polar groups [35]. Nevertheless, in the case of the hydrogenation of nitroarenes with molecular hydrogen, the role played by each type of nitrogen is still unclear, and controversial discussion still arises in the literature [36].

**Scheme 1:**
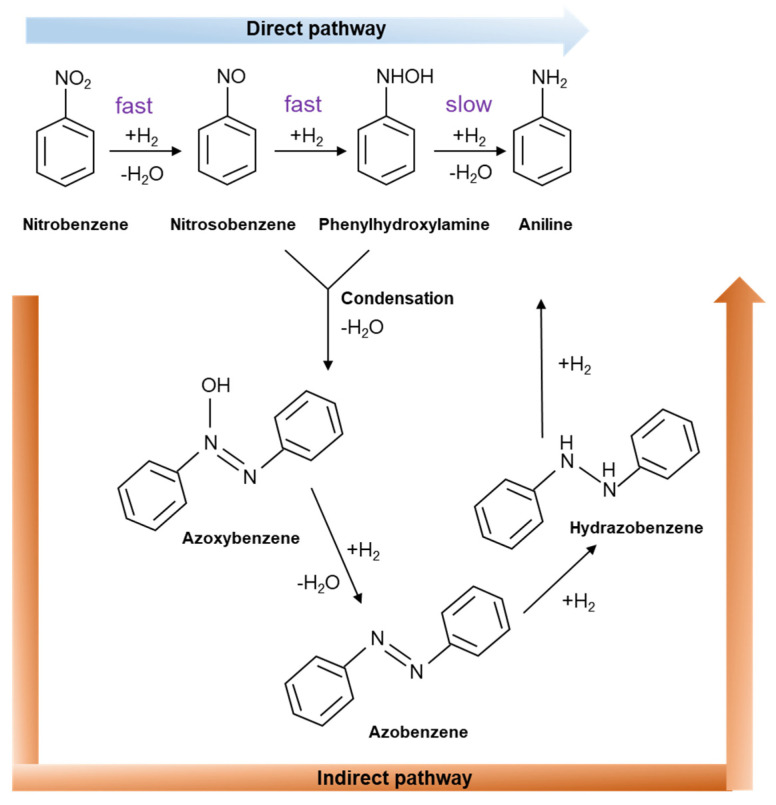
Haber mechanism for the reduction of nitroarenes showing the direct pathway and the indirect (condensation) pathway.

The mechanism of nitrobenzene hydrogenation, first proposed by Haber et al. [13,37,38], includes a direct route and a condensation route, but both include nitrosobenzene as an intermediate [24] (Scheme 1). In the direct pathway, nitrobenzene (PhNO_2_), is reduced to nitrosobenzene (PhNO), which is subsequently reduced to phenylhydroxylamine (PhNHOH) and finally aniline (PhNH_2_). This requires the cleavage of the N-O bond, determining it to be the slow stage of the reaction. If the catalytic activity is not very high, there is a risk of phenylhydroxylamine accumulation leading to an increase in byproducts [39]. 

The indirect pathway progresses through the condensation of nitrosobenzene and phenylhydroxylamine, producing azoxybenzene which will then be reduced to azo, hydrazo and amine compounds in a series of successive steps. The analysis of the intermediates and products of the reaction under different experimental conditions has led to the conclusion that the direct route is preferred to the condensation route unless strong bases are present. However, the actual reaction mechanism is unclear and is still the aim of debate [38,39,40]. The accumulation of intermediate products is a general issue associated with the hydrogenation of nitroarenes. In the particular case of halonitroarenes, there is another added difficulty associated with the production of an undesired mixture of haloanilines and dehalogenation products [41,42]. This makes it even more challenging to find a chemoselective catalyst for the hydrogenation of halonitroarenes, such as 1-chloro-4-nitrobenzene, which is the goal of the present study.

**Scheme 2:**
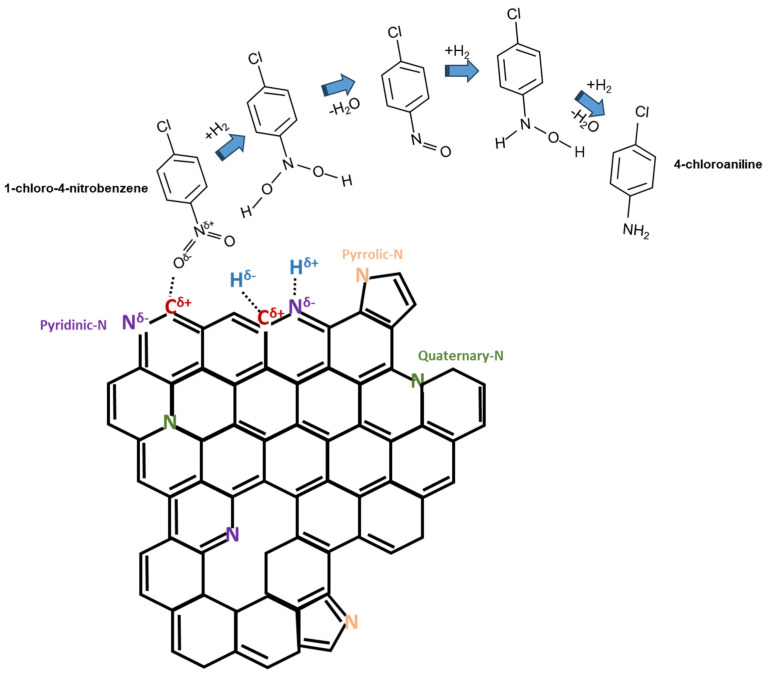
Schematic representation of the mechanism for 1-chloro-4-nitrobenzene reduction on the surface of a N-doped carbon.

H_2_ activation is a critical step in catalytic hydrogenation. This can progress through either homolytic or heterolytic dissociation. The former involves the cleavage of the H-H bond and the formation of two hydrides. When a metal catalyst is present, it can accept electrons from the H_2_ bonding-σ orbital and provide d-electrons to its antibonding-σ* orbital. When H_2_ activation progresses through heterolytic dissociation, H_2_ is cleaved into H^δ+^ and H^δ−^. Irrespective of the mechanism for H_2_ dissociation taking place, usually high activities but extremely low selectivities are achieved with supported metal catalysts [24]. In the search for green and sustainable catalysts with extremely high selectivity for the hydrogenation of the nitro group in the presence of halide groups, metal-free N-doped carbons have been considered in this research work as good candidates for the catalytic reduction of 1-chloro-4-nitrobenzene using benign molecular hydrogen. Charge redistribution in N-doped carbons resulting from the dissimilar electronegativities of C and N favors the adsorption and activation of the nitro (-NO_2_) group and heterolytic cleavage of H_2_. H^δ+^ and H^δ−^ species may then anchor to active sites producing N^δ−^-H^δ+^ and C^δ+^-H^δ−^. Next, the chemoselective reduction of the nitro group is expected to occur with the assistance of N^δ−^-H^δ+^ and C^δ+^-H^δ−^ species. Finally, the desorption of the chloro-aniline product occurs (Scheme 2). 

The benefits of carbon materials as free-metal catalysts depend not only on the presence of heteroatoms but also on adequate textural properties, i.e., high surface area and well-developed porosity, that ease the access of the reactants to the catalytic sites. During carbonization, the volatile content of the raw material is reduced via pyrolysis of the carbon precursors in the presence of an inert gas (N_2_ or Ar) in a temperature range of 300–900 °C [43]. During this pyrolysis treatment, a decomposition process occurs, which leads to the elimination of volatile heteroatoms (mainly hydrogen and oxygen, but also sulfur and nitrogen, among others), in the form of gases such as CO, CO_2_, CH_4_, H_2_, H_2_O, SO_x_ and NO_x_, that produces an enrichment of the material in carbon [44]. Consequently, a primary porosity is created, which in most cases is not enough for catalytic applications. Therefore, a subsequent activation treatment is usually required. The purpose of activation is to improve the specific surface and pore volume of the carbon by the development of the existing porosity and the creation of new pores. However, there is an inherent risk of loss of the doping heteroatom because of the high temperature and long treatment time required for carbon activation.

Different strategies have been developed for the synthesis of nitrogen-doped carbons. Some of them are based on the post-treatment of an already available highly porous activated carbon with a gas such as ammonia, hydrogen cyanide or even aniline vapor that reacts with carbon at a high temperature [45]. Alternatives to the use of toxic reactive gases or vapors [45,46,47,48,49] using safer nitrogen-rich compounds have also been explored. For instance, in our previous studies, pyrrole [50,51], aniline [50,51] and melamine [52] were polymerized on the surface of a commercial mesoporous carbon. The resulting N-doped carbons served as support for Ni, Pt and Co nanoparticles which produced a considerable degree of conversion of palm oil to bio-hydrogenated diesel [50]. They were also successfully used in the hydrodeoxygenation (HDO) of guaiacol [51] and in the hydrogenation of 1-chloro-4-nitrobencene [53]. The main drawback of these procedures falls in the requirement of an existing carbon material with a high surface area and an intrinsic porous structure, which in turn, risks being partially destroyed by the formation of unstable functional groups during the heat treatment, with the consequent deterioration of the carbon properties [54].

Thus, we explored an alternative synthesis strategy based on one-step carbonization and N doping using nitrogen-rich carbon precursors. Instead of our first approach based on the polymerization of pyrrole or aniline monomers on the surface of the activated porous carbon, we synthesized polypyrrole and polyaniline and used the N-rich polymers not only as source of N but also as carbon precursors [52,55]. An adequate control of the experimental variables during the oxidative chemical polymerization of the monomers in aqueous media permitted the tailoring of the structure of the polymeric chain to enhance a homogeneous distribution of nitrogen functionalities, which served as catalytic sites in both the bulk and the carbon surface. However, the resulting polymer-derived carbons exhibited very low surface area and porosity, so an activation treatment was required to maximize the adsorption and catalytic performance of the N-doped carbon materials. Steam activation at 900 °C resulted in an important increase in the surface area and pore development of the polymer-derived carbons, but in turn, considerable decomposition of nitrogen functionalities was produced [52]. Activation treatments require an exhaustive control of time and temperature specifically selected for each carbon material in order to ensure an adequate compromise between well-developed textural properties (surface area, pore volume and pore distribution) and doping degree [55]. It has been reported that in situ doping of the carbon framework tends to form pyrrolic and/or pyridinic N atoms, while graphitic N is normally generated after a high-temperature post-treatment [56]. Nevertheless, the literature shows contradictory results which still raise great controversy. Yet, obtaining an accurate N-doping level in a controlled way in these carbon materials is still a challenge.

In this work, N-doped carbons have been produced using melamine. To overcome the issues associated with an activation treatment, this has been obviated, and instead, calcium citrate has been selected as a porogen agent. For this purpose, mixtures of melamine (C_3_H_6_N_6_) and calcium citrate (C_12_H_10_Ca_3_O_14_) were prepared. Therefore, melamine serves as a carbon precursor and also a source of N, whereas calcium citrate, which decomposes at the pyrolysis temperature, acts not only as a carbon source but also as a pore generator.

The specific surface area and nitrogen content of the N-doped activated carbons were readily tuned by varying the experimental conditions. For that purpose, a series of carbons with different melamine/citrate ratios were synthesized and characterized. Considering the physicochemical and textural properties of the prepared carbons, the melamine/citrate ratio of 2:1 was selected to study the effect of the pyrolysis temperature (from 500 °C to 950 °C) on the nitrogen content and the porosity development. Finally, the catalytic performance of these metal-free N-doped carbons was tested in the reduction of 1-chloro-4-nitrobenzene with molecular hydrogen to selectively produce the corresponding chloro-aniline. 

## 2. Results and Discussion

**Figure 1:**
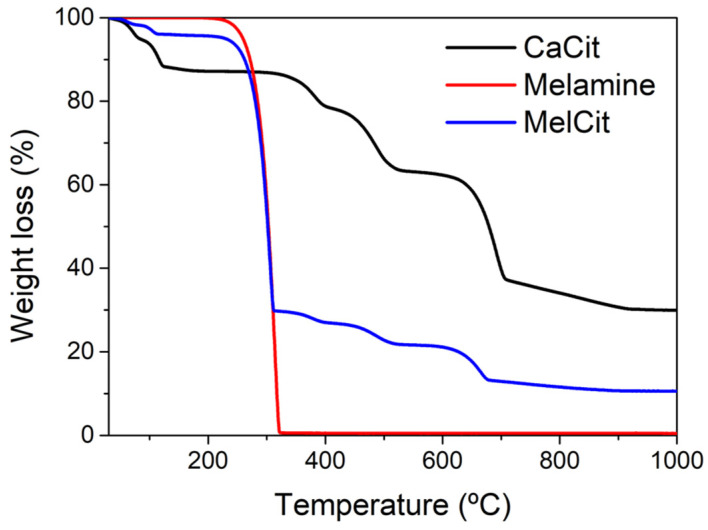
TGA profiles of calcium citrate tetrahydrate, melamine and the corresponding physical mixture of both (2:1 *w*/*w* ratio). Heating rate: 5 °C/min; N_2_ atmosphere.

The carbonization and decomposition processes were monitored using thermogravimetric analysis (TGA). Figure 1 shows the thermogravimetric profile of melamine, calcium citrate and their mixture under N_2_ flow. For calcium citrate, in agreement with previous reports [57,58], five weight loss steps can be appreciated. The first two steps, centered at 65 and 115 °C, respectively, are endothermic, as evaluated by DSC (Figure S1, Supplementary Materials), and correspond to the loss of two water molecules each. The weight losses centered at 300 and 530 °C are assigned to the decomposition of calcium citrate into calcium carbonate, which takes place with the formation and further decomposition of calcium aconitate as an intermediate. Finally, the last weight loss located around 700 °C corresponds to the partial decomposition of CaCO_3_ into CaO, CO and CO_2_ and the formation of the carbon material [53].

**Scheme 3:**
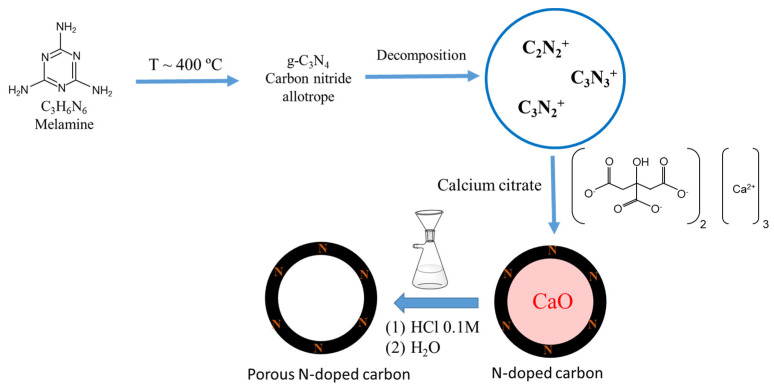
Synthesis of a porous N-doped carbon from the pyrolysis of melamine and calcium citrate.

The TGA profile corresponding to melamine decomposition shows a drastic weight loss starting at around 250 °C, which is accompanied by an endothermic peak, as evaluated by DSC (Figure S1.), centered at 305 °C, which is assigned to the sublimation and/or decomposition of melamine. Finally, the TGA profile of the melamine–citrate mixture is a combination of those of calcium citrate and melamine. It has been reported [59] that at temperatures around 400 °C, melamine polymerizes, yielding a carbon nitride allotrope, g-C_3_N_4_. When the carbonization temperature increases from 400 °C to 950 °C, in the presence of calcium citrate, two processes occur at the same time, the carbonization of calcium citrate and the decomposition of carbon nitride into different reactive nitrogen-containing species such as C_2_N_2_^+^, C_3_N_2_^+^, C_3_N_3_^+^. These species can react with the semi-carbonized calcium citrate to produce a N-doped carbon with a large variety of N-containing functionalities [60] (Scheme 3).

### 2.1. Effect of the Melamine/Citrate Ratio

The surface chemistry of the carbons prepared by pyrolysis of mixtures containing melamine and calcium citrate was analyzed by XPS. Table 1 shows the relative amounts of C, O, N and Ca determined from the corresponding peak areas. All the carbon materials showed the presence of the doping element (nitrogen) in a wide range between 5 and 23 at.%. These results confirm the effectiveness of nitrogen doping using melamine. As expected, the higher the melamine percentage, the higher the amount of nitrogen detected on the surface of the carbon. Ca is detected in some samples due to an uncompleted removal of calcium citrate by the acid-washing treatment in those cases.

**Figure 2:**
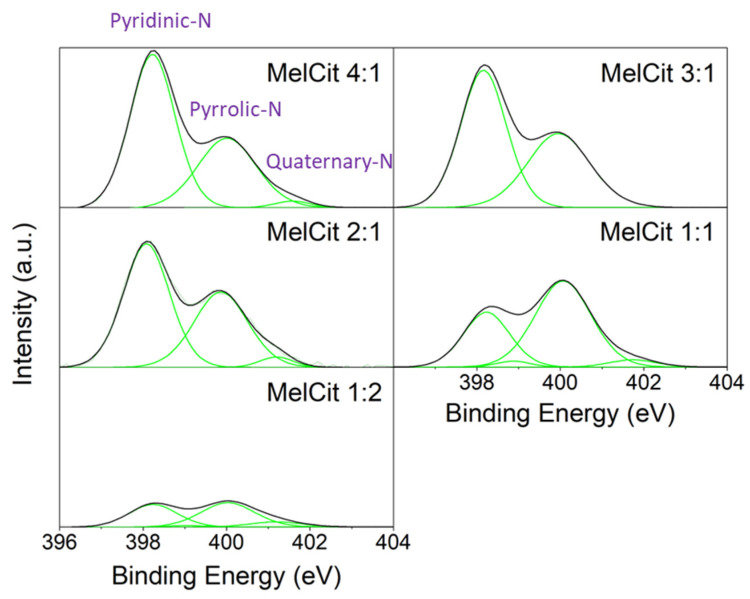
Deconvolution of the N 1s high–resolution XPS spectra.

The nature of the nitrogen functionalities was assessed by the deconvolution of the N 1s high-resolution XPS spectra (Figure 2), and their relative amounts are summarized in Table S1 (Supplementary Materials). Nitrogen functionalities in the form of pyridinic N (centered at 398.1 eV), pyrrolic N (centered at 400.1 eV), quaternary N (also called graphitic N, centered at 401.9 eV) and pyridinic N-oxides (centered at 404.1 eV) are generally found in N-doped carbons [2,59]. In the obtained carbons, N-oxides were not detected in any case. The samples with high melamine ratios showed pyridinic N as the most abundant species, followed by pyrrolic N. Nevertheless, samples with lower melamine contents contained mainly pyrrolic N, followed by pyridinic N. Quaternary N was the less abundant nitrogen functionality in all samples. Each N species differently modifies the electron transport properties of the adjacent C atoms. Pyridinic N substitutes a carbon atom in a six-membered ring and contributes with one p electron to the π system of the sp_2_ carbon matrix. Pyrrolic N substitutes C in a five-ring in a sp_3_ configuration and contributes with two electrons to the π system. Quaternary (graphitic) N substitutes a carbon atom in the hexagonal rings of the sp_2_ carbon plane. Each N functionality is expected to have a role in the catalytic hydrogenation reaction of 1-chloro-4-nitrobenzene. 

**Figure 3:**
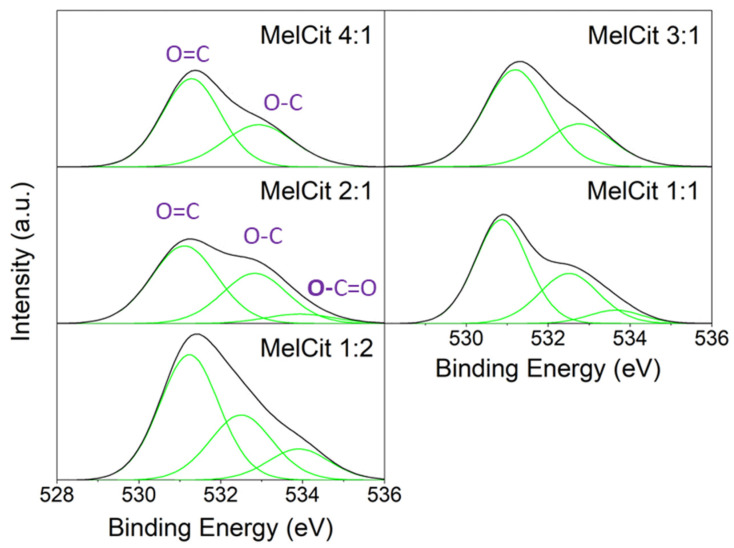
Deconvolution of the O 1s high-resolution XPS spectra.

The amount of oxygen detected by XPS was also affected by the Mel/Cit ratio. As expected, the highest amount of calcium citrate in the precursor mixture (MelCit 1:2) resulted in the highest surface oxygen percentage (12.2 at.% in Table 1). Deconvolution of the O 1s level spectra (Figure 3, Table S2.) shows oxygen species corresponding to O=C at 530.9–531.2 eV, O-C at 532.5–532.9 eV and **O**-C=O at 533.7–533.9 eV [61], which are more evident in the sample with the highest calcium citrate percentage (MelCit 1:2).

**Figure 4:**
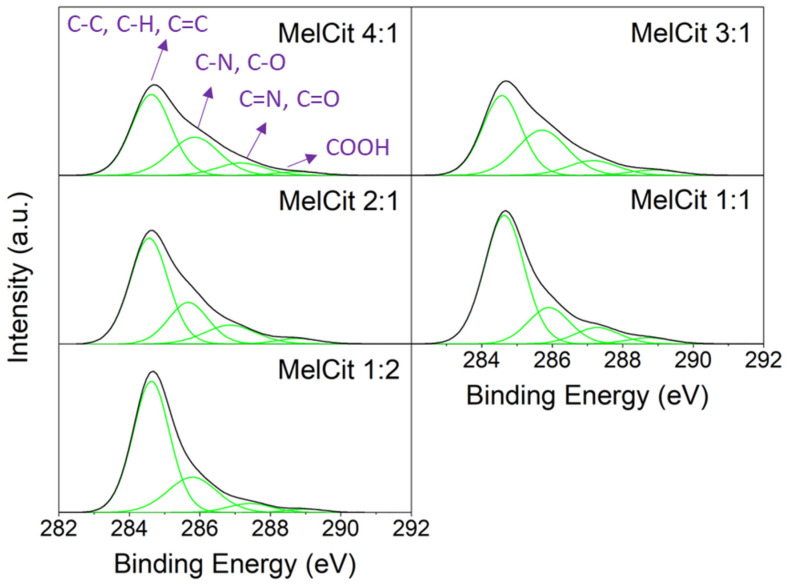
Deconvolution of the C 1s high-resolution XPS spectra.

From the analysis of the C 1s spectra (Figure 4), it is important to remark that the carbon content (at.%) increases as the ratio of calcium citrate increases, which is consequent with calcium citrate being the main carbon source. Four peaks can be identified in the C 1s spectra, which are related to C-C, C-H and C=C (284.6 eV); C-N and C-O (285.6–285.9 eV); C=N and C=O (286.9–287.4 eV); and COOH (288.8–289.0 eV) species. As can be seen in Table S3, there is a decrease in the intensity of the peaks related to carbon bonded with N and O as the amount of calcium citrate increases, which agrees with the survey spectra.

**Figure 5:**
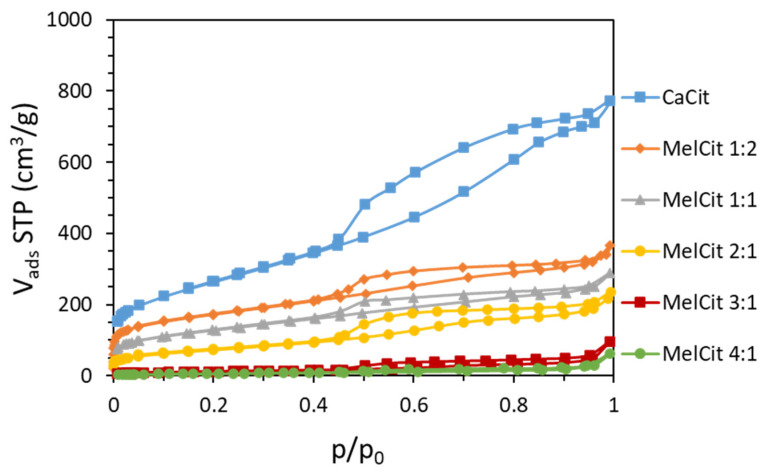
N_2_ adsorption–desorption isotherms at −196 °C of carbon materials prepared with different CaCit and melamine ratios and pyrolyzed at 750 °C.

The textural characterization of the samples was carried out by adsorption experiments using nitrogen. Figure 5 shows that the isotherms are a combination of Type I and Type IV, which are characteristic of microporous–mesoporous solids. The parent carbon obtained from calcium citrate (CaCit) shows a relatively high surface area (949 m^2^/g) and a high volume of mesopores (0.76 cm^3^/g) (Table 2). The nitrogen doping with melamine results in a partial blocking of the pores, which produces a decrease in surface area and porosity. This effect is more evident with the increasing melamine content in the MelCit mixture.

It is evidenced that nitrogen content and textural properties depend to a great extent on the melamine/calcium citrate ratio. Therefore, a balance between the porosity imparted by calcium citrate (evaluated from nitrogen adsorption experiments) and a suitable level of nitrogen doping produced by melamine (assessed by XPS), always ensuring a minimal blocking of the mesopores, has to be reached. Although the sample exhibiting the highest surface area is MelCit 1:2 (611 m^2^/g), this carbon has 5.5 at.% N and 54% mesoporosity (evaluated from the V_meso_/V_total_ ratio). MelCit 1:1 shows less surface area (452 m^2^/g), higher N content (14.1 at.%) and similar mesoporosity (56%). Otherwise, a MelCit ratio of 2:1 provides a carbon with a surface area of 258 m^2^/g, a fairly developed mesoporosity (66%) and a N content of 18.5 at.%.

In the first screening, different melamine/citrate mass ratios were considered, and the experimental results led us to the conclusion that the higher the melamine/calcium citrate ratio, the higher the amount of nitrogen introduced in the carbon matrix. However, there is a potential risk of N functionalities blocking the carbon porosity and negatively affecting the carbon textural properties and catalytic performance, especially when a melamine/calcium citrate ratio higher than 2:1 is used. Then, considering not only the N content, but also taking into account that mesoporosity is a key factor in facilitating the access of the reactants (molecular hydrogen and 1-chloro-4-nitrobenzene) to the catalytic sites, an exhaustive characterization has been carried out on the mesoporous Mel/Cit 2:1 sample, pyrolyzed at different temperatures, to evaluate the mesoporosity, N content, morphology and catalytic activity of this particular sample.

### 2.2. Effect of the Pyrolysis Temperature

The effect of the temperature of pyrolysis on the MelCit 2:1 sample was studied. Pyrolysis was carried out at temperatures ranging from 500 °C to 950 °C, and the physicochemical properties of the samples were determined as described below.

**Figure 6:**
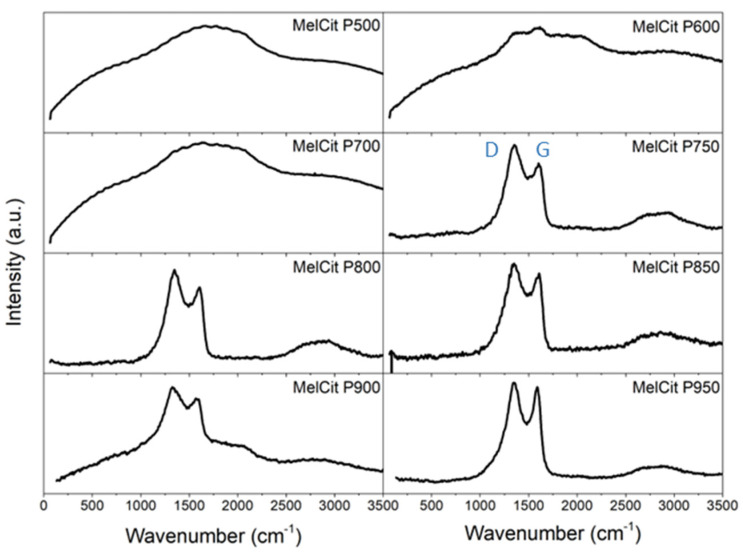
Raman spectra of the carbons obtained after the pyrolysis of MelCit 2:1 mixtures at different temperatures.

Raman spectroscopy allowed the evolution of the carbon structure at different temperatures during the pyrolysis treatment to be followed. G and D bands are characteristic of Raman spectra of activated carbons. The D band is due to the presence of structural defects, whereas the G band is characteristic of graphitic structures and is assigned to the first-order scattering of the symmetric E_2g_ mode of sp^2^ carbon structures [62]. The ratio between the peak intensities (I_D_/I_G_) is a good estimation of the graphitic ordering of the carbon material. The lower the I_D_/I_G_ ratio is, the more graphitic (ordered) the structure is. Pyrolysis treatment at low temperatures (500 to 700 °C) produces amorphous materials, so no D or G bands are observed (Figure 6). Ordering into a graphitic structure starts from 750 °C, as evidenced by the appearance of the D band (1350 cm^−1^) and the G band (1580 cm^−1^). I_D_/I_G_ ratios of 1.09, 1.07, 1.05, 1.03 and 1.02. are obtained for samples pyrolyzed at 750, 800, 850, 900 and 950 °C, respectively. This denotes an increased ordering of the carbon structure with the increase in pyrolysis temperature.

**Figure 7:**
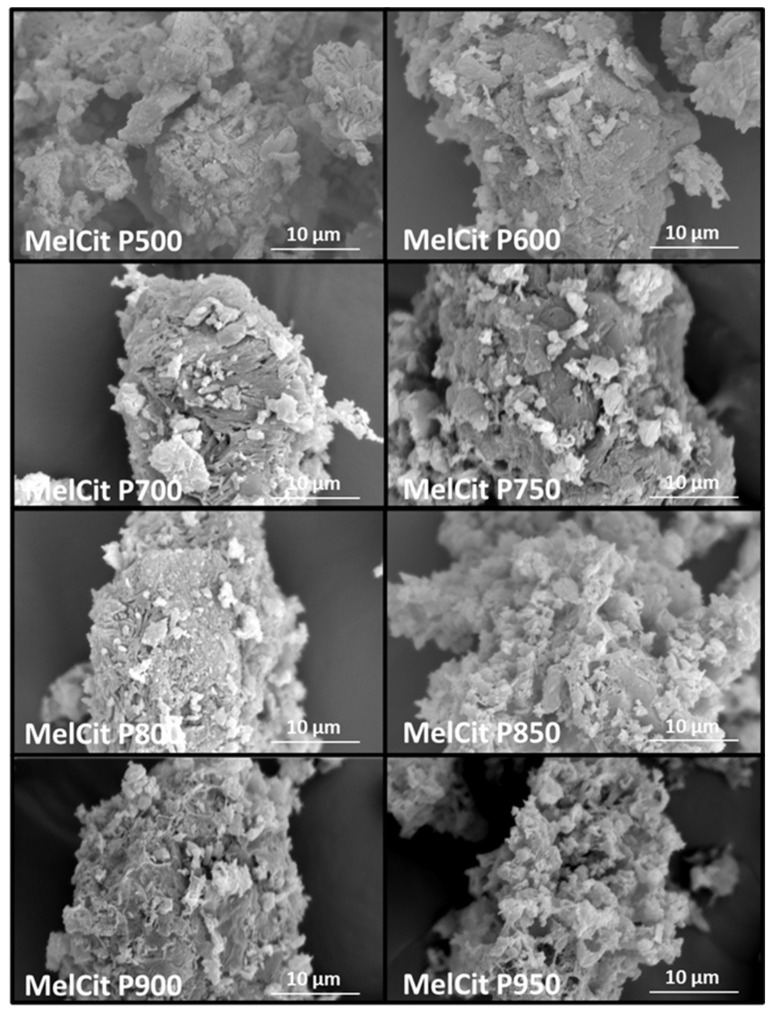
SEM micrographs of MelCit 2:1 carbons obtained after pyrolysis at different temperatures.

As can be observed in the SEM images (Figure 7), the surface texture of the carbons is not greatly affected by the pyrolysis temperature.

**Figure 8:**
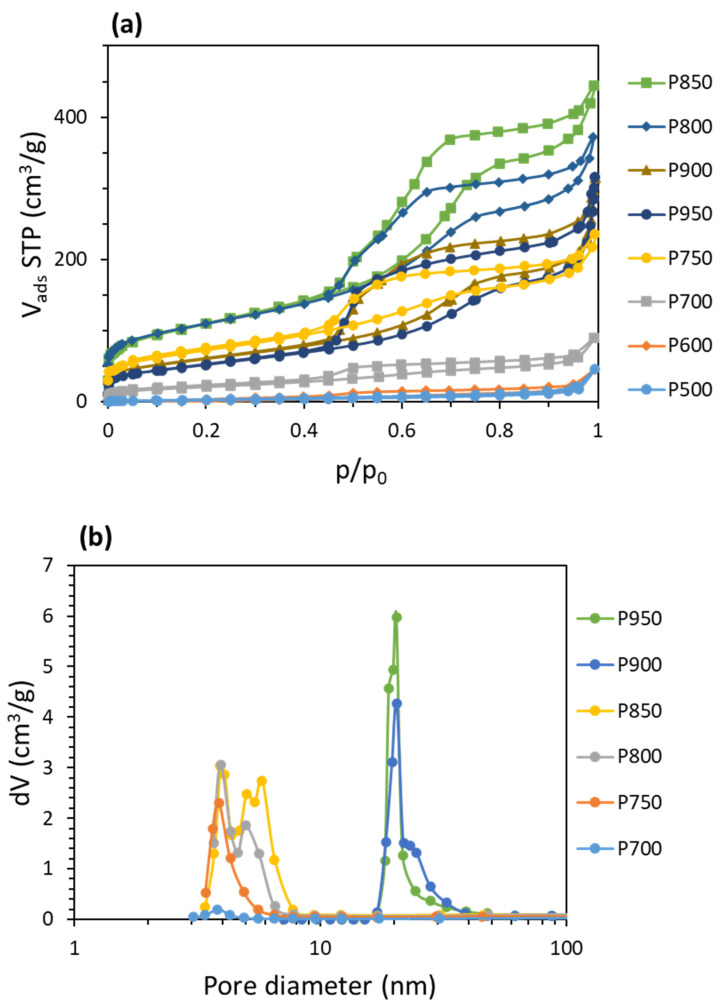
(**a**) N_2_ adsorption–desorption isotherms at −196 °C and (**b**) pore size distribution of the different carbon samples obtained from pyrolysis of MelCit 2:1 sample at different temperatures.

Figure 8 depicts the N_2_ adsorption–desorption isotherms at −196 °C and the pore size distributions of the carbon materials prepared at different pyrolysis temperatures. The corresponding textural parameters are reported in Table 3. The materials obtained after pyrolysis at temperatures lower than 700 °C are nearly non-porous. The treatment at 700 °C produces a material with a small specific surface area (74 m^2^/g). Porosity is clearly developed after pyrolysis at 750 °C, when a carbon with a specific surface area of 258 m^2^/g (Table 3) is obtained. The BET surface of the samples pyrolyzed at 800 and 850 °C increases. Furthermore, there is a clear development of mesoporosity (78%) when pyrolysis is carried out at 850 °C. This yields a carbon with a mesopore volume (0.46 cm^3^/g) which is three and a half times higher than the micropore volume (0.13 cm^3^/g). The pore size distribution obtained by the BJH method (Figure 8b) shows the generation of 4 nm size pores at 750 °C. At 800–850 °C, 7–8 nm pores are created, and there is a clear widening of the mesopores (20 nm) at 900–950 °C. It must be noted that this interesting mesoporous development has been achieved only by a pyrolysis treatment under an inert atmosphere (flowing nitrogen), with no further activation process. However, at high pyrolysis temperatures (900–950 °C), both meso- and microporous volumes decrease (Figure 8a, Table 3), although a widening of the remaining mesopores (20 nm) is produced (Figure 8b). The decrease in surface area may be caused by the formation of unstable functional groups during the heat treatment at T ≥ 900 °C, which partially block the pores, with the consequent deterioration of the carbon textural properties.

**Figure 9:**
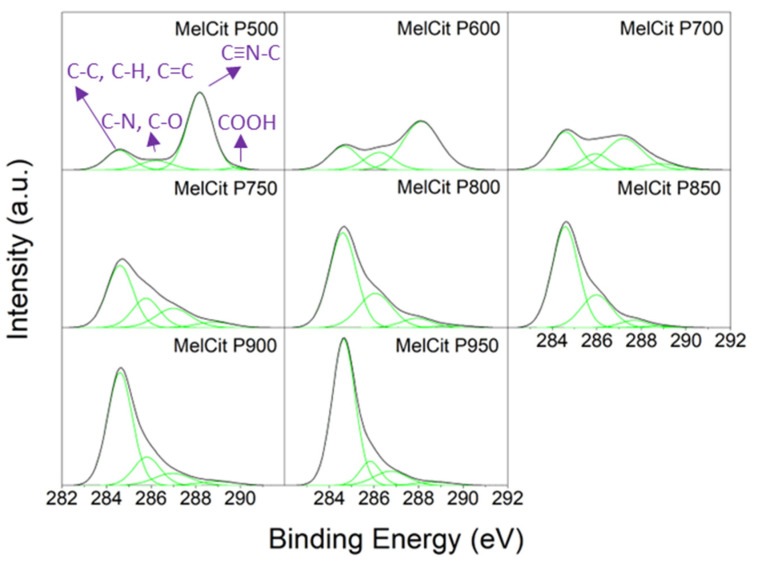
Deconvolution of the C 1s high-resolution XPS spectra of MelCit 2:1 carbons obtained after pyrolysis at different temperatures.

XPS analyses (Table 4) revealed that calcium and oxygen formed by the decomposition of the calcium citrate are present in all the samples, even though they were thoroughly washed. This suggests that calcium oxide is entrapped into the carbon structure (Scheme 3). It is worth noting the increase in the C atomic percentage with the increase in temperature, which agrees with the progress of the carbonization of the raw material during the pyrolysis treatment. At temperatures below 750 °C, a carbon structure is not yet formed, as evidenced by Raman spectroscopy. This can be corroborated by observing the C 1s spectra (Figure 9, Table S4). Thus, the signal for the most abundant carbon species in samples obtained by pyrolysis at low temperatures appears around 288 eV. This peak is assigned to C≡N-C, which is representative of carbon nitride formed during the thermal heating process (Scheme 3).

**Figure 10:**
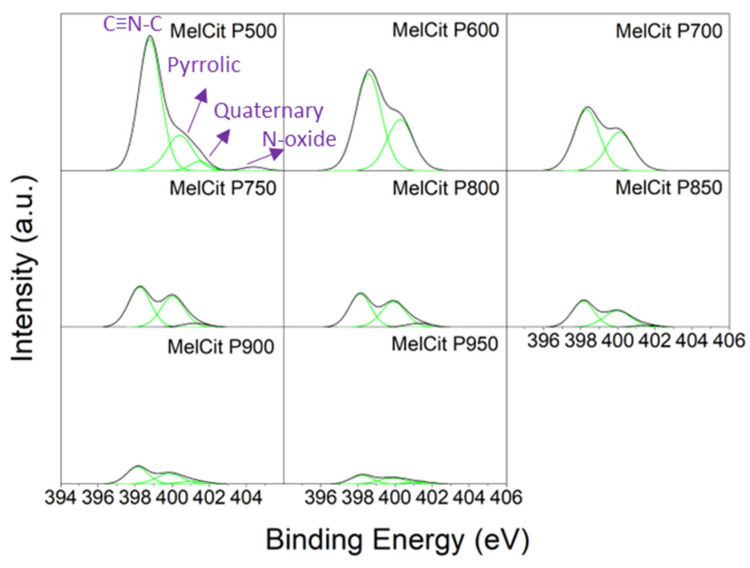
Deconvolution of the N 1s high-resolution XPS spectra of MelCit 2:1 carbons obtained after pyrolysis at different temperatures.

The material pyrolyzed at 500 °C has a very high nitrogen content (49.3 at.%) (Table 4). However, nitrogen is gradually lost at higher pyrolysis temperatures (Figure 10), which is accompanied by a high increase in the carbon percentage. Although nitrogen heteroatoms can be present in the carbon structure in the form of different chemical species (Scheme 2), Table S5 shows a similar distribution of nitrogen functionalities in samples pyrolyzed above 700 °C, with pyridinic N being nearly half of the total N contributions and pyrrolic N being the other half. A much lower contribution of quaternary N is observed, although there is a slight increase in quaternary species in samples pyrolyzed at 900 and 950 °C (11–12% quaternary species; 10.2–6.2% N). These findings differ from data reported in the literature where quaternary N percentages as high as 35% of the total nitrogen (12 at.%) species were reported in samples obtained from mixtures of melamine and chitosan [63]. 

XPS is a surface-sensitive technique, while CHNS elemental analysis provides information on the chemical composition of the bulk. Similar nitrogen percentages were obtained by CHNS (Table 5) and XPS analysis (Table 4). This suggests that a homogeneous dispersion of nitrogen is produced throughout the samples.

**Figure 11:**
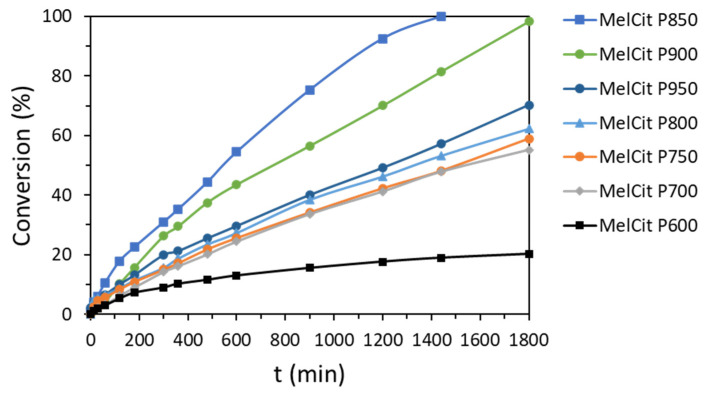
Conversion of 1-chloro-4-nitrobenzene in the catalytic hydrogenation over MelCit 2:1 carbons obtained after pyrolysis at different temperatures.

Our experimental results lead us to the conclusion that the contribution of each N species depends on the melamine/citrate ratio, and also on the pyrolysis and activation experimental conditions. These are highly interesting findings as they bring us the possibility of tuning the desired chemical and textural properties of the N-doped carbons by adequately controlling the preparation experimental conditions. Therefore, it is possible to design the synthesis of a metal-free N-doped catalyst required for a specific application. All the metal-free catalysts based on N-doped carbons obtained from melamine/citrate mixtures in a 2:1 ratio were 100% selective in the hydrogenation of 1-chloro-4-nitrobenzene towards the desired final product, 1-chloro-4-aminobenzene. Intermediate compounds, such as 1-chloro-4-nitrosobenzene and 4,4′- dichloroazoxybenzene, whose presence has been reported [64] in the presence of cobalt catalysts supported on doped carbon materials, were not detected in the presence of any of the metal-free MelCit carbon samples, as shown in the GC-MS analyses (Figures S2–S4.). The best catalytic performances were obtained with the samples pyrolyzed at 850 °C and 900 °C, which produced 100% conversion after 1440 and 1800 min, respectively (Figure 11). Conversions lower than 80% were obtained with the other carbon samples. As expected, the lowest conversion was produced with the sample pyrolyzed at 600 °C, which lacks a carbonaceous structure, as revealed by Raman spectroscopy.

The catalytic performance of the different carbon samples cannot be ascribed only to the electron redistribution on the carbon surface produced by N doping. It is the result of a compromise between the number of active sites and a well-developed mesoporosity, which enhances the access of the reactants to the catalytic sites. The most active catalyst, the MelCit P850 sample (15.4 at.% N), shows the highest total pore volume (0.59 cm^3^/g) and the highest V_meso_ (0.46 cm^3^/g). This happens to be the highest mesoporosity percentage i.e., 78%, evaluated from the V_meso_/V_total_ ratio. The MelCit P800 and MelCit P750 samples (18.5 at.% N) have less developed porosity (0.48 and 0.29 cm^3^/g of total volume, respectively) mainly due to the decrease in the mesopore volume (0.34 and 0.19 cm^3^/g, respectively). In MelCit P900 (10.2 at.% N), there is a clear decrease in mesoporosity (0.25 cm^3^/g mesopore volume), probably due to the blocking of the mesopores with unstable functional groups formed at that high temperature. 

**Figure 12:**
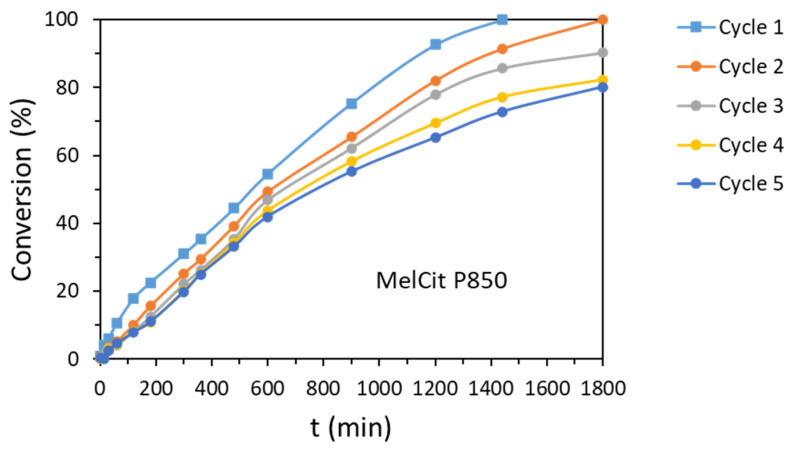
Cyclic experiments showing the reusability of MelCit P850 carbon in the hydrogenation of 1-chloro-4-nitrobenzene.

The reusability of the most active catalyst (MelCit P850) was studied for five subsequent cycles (Figure 12, Table S6). A gradual decrease in conversion from 100% (first cycle) to 80% after five reuses was observed, probably due to the loss of active sites after cyclic tests. However, selectivity was not affected, and the full hydrogenation of the nitro group in the presence of the chlorine functionality was obtained. 

In this work, the effect of temperature in the carbonization of melamine–citrate mixtures has been proven to be an important parameter that defines the catalytic behavior of the prepared metal-free catalysts for the hydrogenation of 1-chloro-4-nitrobenzene, as it affects both the N-doping level and the carbon textural properties. Although the basics of the mechanism of surface reactions associated with catalytic hydrogenation were settled long ago, the issue of low selectivity remains [29]. For instance, Hao et al. [21] proved the synergistic effect of Pd and Au nanoparticles supported on a SiC photocatalyst in the hydrogenation of a variety of nitroarenes to anilines. Several substituted nitroarenes were studied and compared. These nitroarenes were transformed into the corresponding anilines with high activity. However, in particular, *p*-chloronitrobenzene showed low selectivity due to the dehalogenation reaction.

It is well accepted that in order to tune selectivity, it is crucial to control the properties of the catalytic site. This can be achieved either via a careful design of the catalyst surface structure or by modifying its electronic properties by means of surface functionalization. Following the first approach, Zhang et al. [65] prepared carbon nanospheres from xylose using a hydrothermal synthesis and pyrolysis at a high temperature. They concluded that the turbostratic carbon structure formed during the pyrolysis at a high temperature might act as an active center, being able to selectively activate the nitro group in the hydrogenation of *o*-chloronitrobenzene. On the other hand, Liao et al. [63] prepared N-doped carbon materials by the thermal treatment of melamine–chitosan mixtures. Those carbons were active in the hydrogenation of nitrobenzene in hexane, with hydrazine hydrate as a reductant. Their activity was attributed to the presence of quaternary N.

Although there is a clear consensus on the beneficial effect of nitrogen sites in the hydrogenation reaction of nitroarenes, the role of the different nitrogen species as active centers is not clear and is still under debate. Shan et al. [32] prepared a series of metal-free N-doped carbon nanotubes using melanin or protoporphyrin IX as the dopant via a pyrolysis process. The efficient and selective hydrogenation of a variety of substitute nitroarenes containing electron-withdrawing and electron-donating groups at different positions was achieved, and graphitic N was found to play a key role, whereas pyridinic N contributed little to the hydrogenation activity of the catalysts. However, when the halogen group was located at the *para* position of the nitro group, dehalogenation also occurred during the preferential hydrogenation of the nitro group into an amino group, leading to a low selectivity towards chloroaniline (64.3%).

As discussed above, some authors claim that the active sites are quaternary (graphitic) N atoms, but there is not a clear agreement on this. For example, Xiong et al. [36], based on their studies with N-doped carbon nanotubes prepared by post-nitridation, claimed that pyrrolic N atoms work as active sites for nitrobenzene hydrogenation, favoring the chemisorption and dissociation of hydrogen molecules. Nevertheless, their most active catalysts showed conversions of nitrobenzene inferior to 60% and selectivities towards aniline of about 90%. Jarrais et al. [2] studied the catalytic behavior of N-doped carbon nanotubes and graphene flakes in the selective hydrogenation of 4-nitrophenol with NaBH_4_. N doping was achieved by ball milling the carbon materials with melamine, followed by thermal treatment. This resulted in the introduction of pyrrolic, pyridinic and quaternary N atoms on the graphitic structure. Their results showed a good improvement in durability and catalytic activity in the reduction of 4-nitrophenol with NaBH_4_, although conversions were below 80%.

Efficient green catalytic hydrogenation of nitroarenes to anilines is still a challenge. In this work, the use of metal catalysts has been avoided, and the environmentally friendly H_2_ has been used as a reductant instead of NaBH_4_. In addition, the para-halonitroarene 1-chloro-4-nitrobenzene has been chosen on purpose as a target molecule to check the selectivity of the prepared N-doped catalysts for the reduction of the nitro group in the presence of the potentially reducible Cl group. It is worth noting that 100% selectivity was reached in all cases. In addition, the most active catalysts showed 100% conversion after 1440 min (MelCit P850) and 1800 min (MelCit P900). These samples showed mainly pyridinic and pyrrolic species. Nevertheless, our previous studies using metal-free N-doped carbons obtained from polymers showed that the most effective catalysts contained quaternary N functionalities [55]. This leads to the conclusion that more research work is needed to clarify the precise role of nitrogen functionalities in the selective hydrogenation of nitroarenes to the corresponding anilines. Even so, the catalytic activity and selectivity of the prepared carbons undoubtedly cannot be ascribed only to the electron redistribution on the carbon surface produced by N doping. Carbon’s textural properties (surface area, pore size and distribution) have a crucial role in easing the access of the reactants to the catalytic sites. In this research, it has been demonstrated that MelCit P850 carbon, exhibiting a considerable surface area (398 m^2^/g) and a well-developed porosity (V_total_ = 0.59 cm^3^/g; V_mico_ = 0.13 cm^3^/g; V_meso_ = 0.46 cm^3^/g) and containing a high percentage of mesopores (78%, evaluated from the V_meso_/V_total_ ratio), shows the best catalytic performance. This is the consequence of an adequate N-doping level (15.4 at.%) in combination with easy access of the reactants to the catalytic sites through the mesopores.

## 3. Experimental Section

### 3.1. Synthesis of Materials

N-doped carbons were prepared by pyrolysis of homogeneous physical mixtures of melamine (C_3_H_6_N_6_, 2,4,6-triamino-1,3,5-triazine from Sigma Aldrich, Madrid, Spain, 99%) and calcium citrate (Ca_3_C_6_H_5_O_7_·4H_2_O, from Sigma-Aldrich, ≥99%). Several melamine/citrate ratios were considered. Pyrolysis was carried out in a tubular furnace under flowing N_2_ for 1 h. The influence of the pyrolysis temperature was studied between 500 and 950 °C. After the pyrolysis treatment, the samples were treated with a diluted aqueous HCl solution (Sigma-Aldrich, ACS reagent, 37%) to remove the formed CaO and then washed with distilled water. Finally, the carbons were dried at 80 °C for 12 h. Pyrolyzed samples containing different ratios of melamine and calcium citrate were named “MelCit A:B”, with A:B being the mass ratio between melamine and calcium citrate. The samples pyrolyzed at different temperatures were named MelCitP-T, with T being the pyrolysis temperature.

### 3.2. Characterization of Materials

The modifications of the precursor materials during the pyrolysis procedure were followed up by thermogravimetric (TG) analysis using a TGA-DSC 2 instrument (Mettler-Toledo, Cornellà de Llobregat, Barcelona, España). The analyses were performed between 25 and 1000 °C under nitrogen flow (100 mL/min) with a heating rate of 5 °C/min, using an alumina crucible (Mettler-Toledo, Cornellà de Llobregat, Barcelona, España). 

The textural properties of the carbons were determined by N_2_ adsorption isotherms at −196 °C. The isotherms were obtained with an Autosorb-6 instrument. All the samples were previously degassed for 4 h under vacuum at 250 °C in an Autosorb Degasser. Both devices were manufactured by Quantachrome Instruments (Madrid, Spain). 

The morphology of the carbon particles was determined by scanning electron microscopy (SEM) using a Hitachi S3000N instrument (Madrid, Spain) equipped with a Bruker XFlash X-Ray detector 3001 (Madrid, Spain). 

Raman spectroscopy was performed with a Raman Jasco NRS-5100 instrument (Jasco Madrid, Spain) using a 532 mn laser and a 600 lines/mm slit between 0–4000 cm^−1^.

X-ray photoelectron spectroscopy (XPS) was performed using a K-ALPHA spectrometer (ThermoFisher Scientific, Alcobendas, Madrid, España). Mg-K_α_ radiation (1253.6 eV) was used. A twin-crystal monochromator yielded a focused X-ray spot with a diameter of 400 nm, at 3 mA and 12 kV.

Elemental analysis (CHNS) was performed using a microanalyzer equipped with a Micro TruSpec (LECO, Tres Cantos, Madrid, Spain) detection system, using He as carrier gas. This device has infrared and thermal conductivity detectors that simultaneously analyze carbon, hydrogen, nitrogen and sulfur.

### 3.3. Catalytic Tests

The catalytic performance of the prepared materials was tested in the hydrogenation of 1-chloro-4-nitrobenzene using H_2_ as a reductant. Thus, 100 mL of a 0.1 M ethanolic solution of 1-chloro-4-nitrobenzene (ClC_6_H_4_NO_2,_ Sigma Aldrich, 99%), 200 µL of octane (CH_3_(CH_2_)_6_CH_3_, Sigma Aldrich, ≥99%) as an internal standard and 500 mg of carbon (catalyst) were added to a high-pressure stainless-steel reactor (Biometa, Madrid, Spain). The reaction was carried out at a temperature of 150 °C and H_2_ pressure of 50 bar. Aliquots (2 mL) were periodically extracted and analyzed using a gas chromatography device coupled to a mass spectrometer (QP-2010 GC-MS Shimadzu, Alcobendas, Madrid, Spain) and an HP-5 capillary column using helium as carrier gas.

The conversion and selectivity of the carbon samples in the hydrogenation reaction were calculated as follows:

$$Conversion\;\left(\%\right)=100-\frac{\left[CNB\right]}{{\left[CNB\right]}_{0}}*100$$

$$Selectivity\;\left(\%\right)=\frac{\left[CA\right]}{{\left[CNB\right]}_{0}-[CNB]}*100$$

where ${\left[CNB\right]}_{0}$ and $\left[CNB\right]$ are the initial and measured concentrations of 1-chloro-4-nitrobenzene, respectively, and [CA] is the measured concentration of 4-chloroaniline during the progress of the reaction.

Each carbon sample was tested five times in the hydrogenation reaction, and the averages of the conversion data were calculated, with the standard deviation being less than 2% in all cases. A study of the reusability of the most active catalyst was carried out. Thus, the catalyst sample was recovered by filtration after each reaction cycle, washed with ethanol and dried in an oven at 80 °C for 6 h, before being reused for 4 more subsequent cycles.

## 4. Conclusions

Metal-free N-doped carbons were designed and synthesized using melamine as a nitrogen and carbon source and calcium citrate as a carbon source and porogen agent. The influence of the melamine/citrate mass ratio and the temperature during the carbonization of the raw material mixture by a pyrolysis treatment was evaluated. The carbons showed developed porosity, so no further activation treatment was carried out. It is observed that the higher the melamine/calcium citrate ratio, the higher the amount of nitrogen introduced in the carbon matrix. However, there is a potential risk of N functionalities blocking the carbon porosity and negatively affecting the carbon textural properties and catalytic performance when a melamine/calcium citrate ratio higher than 2:1 is used. It is therefore necessary to design the synthesis experimental parameters to achieve an effective N-doping level and at the same time develop mesoporosity to facilitate the access of the reactants (molecular hydrogen and 1-chloro-4-nitrobenzene) to the catalytic sites through the mesopores.

The pyrolysis temperature used to carbonize the melamine–citrate mixture greatly affects the physicochemical properties of the carbon materials. It was observed that a minimum temperature of 750 °C is required to develop a carbonaceous structure. The increase in the pyrolysis temperature up to 850 °C is beneficial for developing mesoporosity and surface area, but a further increase in the pyrolysis temperature (900–950 °C) produces the deterioration of the porous structure and a drastic loss of N functionalities.

The synthesized metal-free N-doped carbons were tested in the reduction of 1-chloro-4-nitrobenzene with molecular hydrogen. Full selectivity towards the hydrogenation of the nitro group was obtained in all cases. The best performance (full conversion and full selectivity) was achieved with the catalyst prepared from a 2:1 melamine–citrate mixture pyrolyzed at 850 °C, which showed not only an adequate doping level, but also a well-developed mesoporous structure that enhances the access of the reactants to the catalytic sites and prevents diffusion issues associated with micropores.

In this work, it has been demonstrated that it is possible to design highly efficient metal-free catalysts based on N-doped carbons obtained from melamine–citrate mixtures by controlling the relative amount of the precursors and the preparation conditions. Future research work will be aimed at the design of new active and selective metal-free catalysts for the hydrogenation of nitroarenes which include a variety of doping heteroatoms.

## Figures and Tables

**Table 1:** XPS surface composition (at.%) for the carbons obtained by pyrolysis of melamine–citrate mixtures at 750 °C.

Sample	C 1s	O 1s	N 1s	Ca 2p_3/2_
MelCit 4:1	69.1	7.8	23.1	-
MelCit 3:1	69.1	7.8	20.6	2.5
MelCit 2:1	72.0	7.7	18.5	1.8
MelCit 1:1	78.0	7.6	14.1	0.3
MelCit 1:2	79.8	12.2	5.5	2.5

**Table 2:** Textural properties obtained from the N_2_ adsorption–desorption isotherms at –196 °C for several carbon materials.

Sample	V_total_ (cm^3^/g)	V_micro_ (cm^3^/g)	V_meso_ (cm^3^/g)	BET Surface Area (m^3^/g)
MelCit 4:1	0.05	0.01	0.04	25
MelCit 3:1	0.07	0.02	0.05	43
MelCit 2:1	0.29	0.10	0.19	258
MelCit 1:1	0.39	0.17	0.22	452
MelCit 1:2	0.50	0.23	0.27	611
CaCit	1.10	0.34	0.76	949

**Table 3:** Textural properties obtained from the N_2_ adsorption–desorption isotherms at –196 °C for the MelCit 2:1 carbon material pyrolyzed at different temperatures.

Sample	V_total_ (cm^3^/g)	V_micro_ (cm^3^/g)	V_meso_ (cm^3^/g)	BET Surface Area (m^2^/g)
MelCit P500	0.03	0.02	0.01	0
MelCit P600	0.03	0.00	0.03	11
MelCit P700	0.09	0.02	0.07	74
MelCit P750	0.29	0.10	0.19	258
MelCit P800	0.48	0.14	0.34	388
MelCit P850	0.59	0.13	0.46	398
MelCit P900	0.33	0.08	0.25	213
MelCit P950	0.31	0.07	0.24	188

**Table 4:** XPS surface composition (at.%) for the carbons obtained after pyrolysis at different temperatures.

Sample	C 1s	O 1s	N 1s	Ca 2p_3/2_
MelCit P500	47.4	3.2	49.3	0.1
MelCit P600	49.0	5.7	44.9	0.5
MelCit P700	57.0	6.2	35.9	1.0
MelCit P750	72.0	7.7	18.5	1.8
MelCit P800	74.6	6.0	18.6	0.9
MelCit P850	78.1	4.9	15.4	1.6
MelCit P900	81.6	6.5	10.2	1.8
MelCit P950	89.0	3.8	6.2	1.0

**Table 5:** Atomic composition of the carbon surfaces obtained by CHNS.

Sample	C (%)	H (%)	N (%)
MelCit P500	40.37	2.58	41.46
MelCit P600	44.10	2.37	31.95
MelCit P700	45.95	2.01	28.89
MelCit P750	53.07	2.20	21.41
MelCit P800	50.38	1.71	20.14
MelCit P850	59.77	1.38	14.87
MelCit P900	63.72	1.28	10.26
MelCit P950	72.96	1.35	7.64

## Data Availability

Data are available on request from the authors.

## Supplementary Materials

The following supporting information can be downloaded at https://www.mdpi.com/article/10.3390/ijms25052515/s1.

## References

[1] Zhang G., Wang L., Shen K., Zhao D., Freeman H.S. (2008). Hydrogenation of O-Chloronitrobenzene on a Pd/C Catalyst Doped with Metal Oxide Nanoparticles. Chem. Eng. J..

[2] Jarrais B., Guedes A., Freire C. (2018). Heteroatom-Doped Carbon Nanomaterials as Metal-Free Catalysts for the Reduction of 4-Nitrophenol. ChemistrySelect.

[3] Kovacic P., Somanathan R. (2014). Nitroaromatic Compounds: Environmental Toxicity, Carcinogenicity, Mutagenicity, Therapy and Mechanism. J. Appl. Toxicol..

[4] Movahed S.K., Jafari P., Mallakpour S. (2023). Ruthenium Nickel Bimetallic Nanoparticles Embedded in Nitrogen-Doped Carbon Mesoporous Spheres as a Superior Catalyst for the Hydrogenation of Toxic Nitroarenes. J. Environ. Chem. Eng..

[5] Béchamp A. (1854). De l’action des protosels de fer sur la nitronaphtaline et la nitrobenzine: Nouvelle méthode de formation des bases orgnaiques artificielles de zinin. Ann. Chim. Phys..

[6] Lara P., Philippot K. (2014). The Hydrogenation of Nitroarenes Mediated by Platinum Nanoparticles: An Overview. Catal. Sci. Technol..

[7] Wienhöfer G., Sorribes I., Boddien A., Westerhaus F., Junge K., Junge H., Llusar R., Beller M. (2011). General and Selective Iron-Catalyzed Transfer Hydrogenation of Nitroarenes without Base. J. Am. Chem. Soc..

[8] Jagadeesh R.V., Banerjee D., Arockiam P.B., Junge H., Junge K., Pohl M.M., Radnik J., Brückner A., Beller M. (2015). Highly Selective Transfer Hydrogenation of Functionalised Nitroarenes Using Cobalt-Based Nanocatalysts. Green Chem..

[9] Ghosh S.K., Mandal M., Kundu S., Nath S., Pal T. (2004). Bimetallic Pt-Ni Nanoparticles Can Catalyze Reduction of Aromatic Nitro Compounds by Sodium Borohydride in Aqueous Solution. Appl. Catal. A Gen..

[10] Liu X., Cheng H., Cui P. (2014). Catalysis by Silver Nanoparticles/Porous Silicon for the Reduction of Nitroaromatics in the Presence of Sodium Borohydride. Appl. Surf. Sci..

[11] Jagadeesh R.V., Wienhöfer G., Westerhaus F.A., Surkus A.E., Pohl M.M., Junge H., Junge K., Beller M. (2011). Efficient and Highly Selective Iron-Catalyzed Reduction of Nitroarenes. Chem. Commun..

[12] Sun X., Olivos-Suarez A.I., Osadchii D., Romero M.J.V., Kapteijn F., Gascon J. (2018). Single Cobalt Sites in Mesoporous N-Doped Carbon Matrix for Selective Catalytic Hydrogenation of Nitroarenes. J. Catal..

[13] Blaser H.U., Steiner H., Studer M. (2009). Selective Catalytic Hydrogenation of Functionalized Nitroarenes: An Update. ChemCatChem.

[14] Wienhöfer G., Baseda-Krüger M., Ziebart C., Westerhaus F.A., Baumann W., Jackstell R., Junge K., Beller M. (2013). Hydrogenation of Nitroarenes Using Defined Iron-Phosphine Catalysts. Chem. Commun..

[15] Reis P.M., Royo B. (2009). Chemoselective Hydrogenation of Nitroarenes and Deoxygenation of Pyridine N-Oxides with H_2_ Catalyzed by MoO_2_Cl_2_. Tetrahedron Lett..

[16] Corma A., González-Arellano C., Iglesias M., Sánchez F. (2009). Gold Complexes as Catalysts: Chemoselective Hydrogenation of Nitroarenes. Appl. Catal. A Gen..

[17] Nie R., Wang J., Wang L., Qin Y., Chen P., Hou Z. (2012). Platinum Supported on Reduced Graphene Oxide as a Catalyst for Hydrogenation of Nitroarenes. Carbon.

[18] Liu Y., Fang Y., Lu X., Wei Z., Li X. (2013). Hydrogenation of nitrobenzene to p-aminophenol using Pt/C catalyst and carbon-based solid acid. Chem. Eng. J..

[19] Takasaki M., Motoyama Y., Higashi K., Yoon S.H., Mochida I., Nagashima H. (2008). Chemoselective Hydrogenation of Nitroarenes with Carbon Nanofiber-Supported Platinum and Palladium Nanoparticles. Org. Lett..

[20] Pothu R., Mitta H., Banerjee P., Boddula R., Srivastava R.K., Kalambate P.K., Naik R., Radwan A.B., Al-Qahtani N. (2023). Insights into the influence of Pd loading on CeO_2_ catalysts for CO_2_ hydrogenation to methanol. Mater. Sci. Energy Technol..

[21] Hao C.-H., Guo X.-N., Sankar M., Yang H., Ma B., Zhang Y.-F., Tong X.-L., Jin G.-Q., Guo X.-Y. (2018). Synergistic Effect of Segregated Pd and Au Nanoparticles on Semiconducting SiC for Efficient Photocatalytic Hydrogenation of Nitroarenes. ACS Appl. Mater. Interfaces.

[22] Yue S., Wang X., Li S., Sheng Y., Zou X., Lu X., Zhang C. (2020). Highly Selective Hydrogenation of Halogenated Nitroarenes over Ru/CN Nanocomposites by In Situ Pyrolysis. New J. Chem..

[23] Dongil A.B., Rivera-Cárcamo C., Pastor-Pérez L., Sepúlveda-Escribano A., Reyes P. (2015). Ir Supported over Carbon Materials for the Selective Hydrogenation of Chloronitrobenzenes. Catal. Today.

[24] Hu Z.N., Liang J., Ding K., Ai Y., Liang Q., Sun H. (2021). bin Insight into the Selectivity of Nano-Catalytic Nitroarenes Reduction over Other Active Groups by Exploring Hydrogen Sources and Metal Components. Appl. Catal. A Gen..

[25] Xu Q., Liu X.M., Chen J.R., Li R.X., Li X.J. (2006). Modification Mechanism of Sn_4_+ for Hydrogenation of P-Chloronitrobenzene over PVP-Pd/γ-Al_2_O_3_. J. Mol. Catal. A Chem..

[26] Jiang C., Shang Z., Liang X. (2015). Chemoselective Transfer Hydrogenation of Nitroarenes Catalyzed by Highly Dispersed, Supported Nickel Nanoparticles. ACS Catal..

[27] Cheong W.C., Yang W., Zhang J., Li Y., Zhao D., Liu S., Wu K., Liu Q., Zhang C., Wang D. (2019). Isolated Iron Single-Atomic Site-Catalyzed Chemoselective Transfer Hydrogenation of Nitroarenes to Arylamines. ACS Appl. Mater. Interfaces.

[28] Pothu R., Challa P., Rajesh R., Boddula R., Balaga R., Balla P., Perugopu V., Radwan A.B., Abdullah A.M., Al-Qahtani N. (2022). Vapour-Phase Selective Hydrogenation of -Valerolactone to 2-Methyltetrahydrofuran Biofuel over Silica-Supported Copper Catalysts. Nanomaterials.

[29] Zaera F. (2017). The Surface Chemistry of Metal-Based Hydrogenation Catalysis. ACS Catal..

[30] Zhuang X., Jin K., Zhang Q., Liu J., Zhang X., Zhan H., Ma L. (2023). One-Pot Synthesis of FexOy Nanoparticles Embedded within N-Doped Carbon Layers as Highly Efficient and Selective Catalysts for the Hydrogenation of Nitroarenes. Chin. Chem. Lett..

[31] Yang F., Chi C., Wang C., Wang Y., Li Y. (2016). High Graphite N Content in Nitrogen-Doped Graphene as an Efficient Metal-Free Catalyst for Reduction of Nitroarenes in Water. Green Chem..

[32] Shan J., Sun X., Zheng S., Wang T., Zhang X., Li G. (2019). Graphitic N-Dominated Nitrogen-Doped Carbon Nanotubes as Efficient Metal-Free Catalysts for Hydrogenation of Nitroarenes. Carbon.

[33] Wei Q., Tong X., Zhang G., Qiao J., Gong Q., Sun S. (2015). Nitrogen-Doped Carbon Nanotube and Graphene Materials for Oxygen Reduction Reactions. Catalysts.

[34] Chacón F.J., Cayuela M.L., Roig A., Sánchez-Monedero M.A. (2017). Understanding, Measuring and Tuning the Electrochemical Properties of Biochar for Environmental Applications. Rev. Environ. Sci. Biotechnol..

[35] Bubanale S., Shivashankar M. (2017). History, Method of Production, Structure and Applications of Activated Carbon. Int. J. Eng. Res. Technol. (IJERT).

[36] Xiong W., Wang Z., He S., Hao F., Yang Y., Lv Y., Zhang W., Liu P., Luo H. (2020). Nitrogen-Doped Carbon Nanotubes as a Highly Active Metal-Free Catalyst for Nitrobenzene Hydrogenation. Appl. Catal. B.

[37] Serna P., Corma A. (2015). Transforming Nano Metal Nonselective Particulates into Chemoselective Catalysts for Hydrogenation of Substituted Nitrobenzenes. ACS Catal..

[38] Wang H., Zhang W., Liu Y., Pu M., Lei M. (2021). First-Principles Study on the Mechanism of Nitrobenzene Reduction to Aniline Catalyzed by a N-Doped Carbon-Supported Cobalt Single-Atom Catalyst. J. Phys. Chem. C.

[39] Chen M., Zhang M., Liu X., Liu Y. (2023). Pt/C-Catalyzed Selective Hydrogenation of Nitroarenes to N-Arylhydroxylamines under Mild Conditions. ChemistrySelect.

[40] Zuo L.J., Xue K.Z., Xu S.L., Kong Y., Liang H.W. (2023). Intermetallic Rh3Sn2 Catalysts for Selective Hydrogenation of Functionalized Nitroarenes to Aromatic Ammonia. ChemCatChem.

[41] Liu X., Wang C., Meng J., Yue X., Wang Q., Lu J., Wang J., Wang X., Zong Y., Jiang X. (2023). Single-Atom Cobalt Catalysts for Chemoselective Hydrogenation of Nitroarenes to Anilines. Chin. Chem. Lett..

[42] Sheng Y., Wu B., Ren J., Wang X., Zou X., Lu X. (2022). Efficient and Recyclable Bimetallic Co–Cu Catalysts for Selective Hydrogenation of Halogenated Nitroarenes. J. Alloys Compd..

[43] Gao Y., Yue Q., Gao B., Li A. (2020). Insight into Activated Carbon from Different Kinds of Chemical Activating Agents: A Review. Sci. Total Environ..

[44] Sekirifa M.L., Hadj-Mahammed M., Pallier S., Baameur L., Richard D., Al-Dujaili A.H. (2013). Preparation and Characterization of an Activated Carbon from a Date Stones Variety by Physical Activation with Carbon Dioxide. J. Anal. Appl. Pyrolysis.

[45] Zhao A., Masa J., Schuhmann W., Xia W. (2013). Activation and Stabilization of Nitrogen-Doped Carbon Nanotubes as Electrocatalysts in the Oxygen Reduction Reaction at Strongly Alkaline Conditions. J. Phys. Chem. C.

[46] Horikawa T., Sakao N., Sekida T., Hayashi J., Do D.D., Katoh M. (2012). Preparation of Nitrogen-Doped Porous Carbon by Ammonia Gas Treatment and the Effects of N-Doping on Water Adsorption. Carbon.

[47] Luo W., Wang B., Heron C.G., Allen M.J., Morre J., Maier C.S., Stickle W.F., Ji X. (2014). Pyrolysis of Cellulose under Ammonia Leads to Nitrogen-Doped Nanoporous Carbon Generated through Methane Formation. Nano Lett..

[48] Skudin V., Andreeva T., Myachina M., Gavrilova N. (2022). CVD-Synthesis of N-CNT Using Propane and Ammonia. Materials.

[49] Hasegawa G., Deguchi T., Kanamori K., Kobayashi Y., Kageyama H., Abe T., Nakanishi K. (2015). High-Level Doping of Nitrogen, Phosphorus, and Sulfur into Activated Carbon Monoliths and Their Electrochemical Capacitances. Chem. Mater..

[50] Jin W., Pastor-Pérez L., Villora-Pico J.J., Pastor-Blas M.M., Sepúlveda-Escribano A., Gu S., Charisiou N.D., Papageridis K., Goula M.A., Reina T.R. (2019). Catalytic Conversion of Palm Oil to Bio-Hydrogenated Diesel over Novel N-Doped Activated Carbon Supported Pt Nanoparticles. Energies.

[51] Jin W., Pastor-Pérez L., Villora-Picó J.J., Pastor-Blas M.M., Odriozola J.A., Sepúlveda-Escribano A., Reina T.R. (2021). In-Situ HDO of Guaiacol over Nitrogen-Doped Activated Carbon Supported Nickel Nanoparticles. Appl. Catal. A Gen..

[52] Villora-Picó J.J., Pastor-Blas M.M., Sepúlveda-Escribano A. (2022). N-Doped Activated Carbons from Polypyrrole—Effect of Steam Activation Conditions. Chem. Ing. Tech..

[53] Villora-Picó J.J., Campello-Gómez I., Serrano-Ruiz J.C., Pastor-Blas M.M., Sepúlveda-Escribano A., Ramos-Fernández E.V. (2021). Hydrogenation of 4-Nitrochlorobenzene Catalysed by Cobalt Nanoparticles Supported on Nitrogen-Doped Activated Carbon. Catal. Sci. Technol..

[54] An B., Xu S., Li L., Tao J., Huang F., Geng X. (2013). Carbon Nanotubes Coated with a Nitrogen-Doped Carbon Layer and Its Enhanced Electrochemical Capacitance. J. Mater. Chem. A Mater..

[55] Villora-Picó J.-J., Gil-Muñoz G., Sepúlveda-Escribano A., Pastor-Blas M.M. (2024). Doped Activated Carbons Obtained from Nitrogen and Sulfur-Containing Polymers as Metal-Free Catalysts for Application in Nitroarenes Hydrogenation. Int. J. Hydrogen Energy.

[56] Sharifi T., Hu G., Jia X., Wågberg T. (2012). Formation of Active Sites for Oxygen Reduction Reactions by Transformation of Nitrogen Functionalities in Nitrogen-Doped Carbon Nanotubes. ACS Nano.

[57] Mansour S.A.A. (1994). Thermal Decomposition of Calcium Citrate Tetrahydrate. Thermochim. Acta.

[58] Zhou Q.Q., Chen X.Y., Wang B. (2012). An Activation-Free Protocol for Preparing Porous Carbon from Calcium Citrate and the Capacitive Performance. Microporous Mesoporous Mater..

[59] Fuertes A.B., Ferrero G.A., Sevilla M. (2014). One-Pot Synthesis of Microporous Carbons Highly Enriched in Nitrogen and Their Electrochemical Performance. J. Mater. Chem. A.

[60] Thomas A., Fischer A., Goettmann F., Antonietti M., Müller J.O., Schlögl R., Carlsson J.M. (2008). Graphitic Carbon Nitride Materials: Variation of Structure and Morphology and Their Use as Metal-Free Catalysts. J. Mater. Chem..

[61] Chiang Y.C., Yeh C.Y., Weng C.H. (2019). Carbon Dioxide Adsorption on Porous and Functionalized Activated Carbon Fibers. Appl. Sci..

[62] Ferrari A.C., Robertson J. (2000). Interpretation of Raman Spectra of Disordered and Amorphous Carbon. Phys. Rev. B.

[63] Liao C., Liu B., Chi Q., Zhang Z. (2018). Nitrogen-Doped Carbon Materials for the Metal-Free Reduction of Nitro Compounds. ACS Appl. Mater. Interfaces.

[64] Duran-Uribe E.D., Sepúlveda-Escribano A., Ramos-Fernández E.V. (2023). Microwave-assisted synthesis of CoxP@C catalysts: Application to the hydrogenation of 1-chloro-4-nitrobezene. Chem. Eng. J..

[65] Zhang P., Song X., Yu C., Gui J., Qiu J. (2017). Biomass-Derived Carbon Nanospheres with Turbostratic Structure as Metal-Free Catalysts for Selective Hydrogenation of o-Chloronitrobenzene. ACS Sustain. Chem. Eng..

