# Supplementary material for: Design and Synthesis of N-Doped Carbons as Efficient Metal-Free Catalysts in the Hydrogenation of 1-Chloro-4-Nitrobenzene
Source: Int J Mol Sci. 2024 Feb 21;25(5):2515. doi: 10.3390/ijms25052515 (PMC10931987; doi:10.3390/ijms25052515)
Supplement: Supplementary file 1 [file ijms-25-02515-s001.zip › ijms-2849661- supplementary materials.pdf]

International Journal of *Molecular Sciences*

MDPI

Article

Design and Synthesis of N-Doped Carbons as Efficient Metal-Free Catalysts in the Hydrogenation of 1-Chloro-4-Nitrobenzene

Juan-José Villora-Picó, Antonio Sepúlveda-Escribano and M. Mercedes Pastor-Blas *

Laboratory of Advanced Materials, Department of Inorganic Chemistry—University Institute of Materials of Alicante, University of Alicante, P.O. Box 99, E-03080 Alicante, Spain
* Correspondence: M. Mercedes Pastor-Blas E-mail address: mercedes.pastor@ua.es_Phone: +34-965903400.

Supplementary Materials:

Table S1. Relative percentages obtained from the deconvolution of the XPS high resolution N1s spectra of melamine-citrate carbons obtained after pyrolysis at 750 °C of different mixtures of melamine-citrate.

Sample	Energy (eV)	Species	at. %	%
**MelCit 4:1**	398.22	Pyridinic	14.27	62
	400.01	Pyrrolic	8.37	36
	401.58	Quaternary	0.48	2
**MelCit 3:1**	398.16	Pyridinic	11.86	58
	399.96	Pyrrolic	8.72	42
**MelCit 2:1**	398.24	Pyridinic	13.23	52
	400.01	Pyrrolic	10.72	42
	401.23	Quaternary	1.50	6
**MelCit 1:1**	398.23	Pyridinic	4.92	35
	400.07	Pyrrolic	8.61	61
	401.74	Quaternary	0.61	4
**MelCit 1:2**	398.24	Pyridinic	2.31	42
	400.03	Pyrrolic	2.60	48
	401.18	Quaternary	0.54	10

Table S2. Relative percentages obtained from the deconvolution of the XPS high resolution O 1s spectra of melamine-citrate carbons obtained after pyrolysis at 750 °C of different mixtures of melamine-citrate.

Sample	Energy (eV)	Species	at. %	%
**MelCit 4:1**	531.28	O=C	4.91	64
	532.93	O-C	2.82	36
**MelCit 3:1**	531.20	O=C	5.28	68
	532.76	O-C	2.49	32
**MelCit 2:1**	531.11	O=C	4.33	57
	532.83	O-C	2.79	36
	533.93	O-C=O	0.56	7
**MelCit 1:1**	530.86	O=C	4.52	60
	532.51	O-C	2.47	32
	533.65	O-C=O	0.59	8
**MelCit 1:2**	531.23	O=C	6.78	56
	532.50	O-C	3.71	30
	533.91	O-C=O	1.75	14

Table S3. Relative percentages obtained from the deconvolution of the XPS high resolution C 1s spectra of melamine-citrate carbons obtained after pyrolysis at 750 °C of different mixtures of melamine-citrate.

Sample	Energy (eV)	Species	at. %	%
**MelCit 4:1**	284.62	C-C, C-H, C=C	37.83	55
	285.85	C-N, C-O	22.15	32
	287.17	C=N, C=O	7.13	10
	288.78	COOH	2.03	3
**MelCit 3:1**	284.57	C-C, C-H, C=C	33.62	49
	285.71	C-N, C-O	24.53	35
	287.15	C=N, C=O	8.01	12
	288.9	COOH	2.98	4
**MelCit 2:1**	284.60	C-C, C-H, C=C	33.65	49
	285.77	C-N, C-O	16.53	24
	286.99	C=N, C=O	14.03	21
	288.84	COOH	4.22	6
**MelCit 1:1**	284.64	C-C, C-H, C=C	52.45	67
	285.91	C-N, C-O	15.38	20
	287.28	C=N, C=O	7.39	9
	288.73	COOH	2.81	4
**MelCit 1:2**	284.62	C-C, C-H, C=C	54.14	68
	285.78	C-N, C-O	19.71	25
	287.42	C=N, C=O	4.44	5
	289.00	COOH	1.54	2

Table S4. Relative percentages obtained from the deconvolution of the XPS high resolution C 1s spectra of MelCit 2:1 carbons obtained after pyrolysis at different temperatures.

Sample	Energy (eV)	Species	at. %	%
**MelCit P500**	284.56	C-C, C-H, C=C	8.76	18
	286.22	C-N, C-O	5.07	11
	288.18	C≡N-C	32.91	69
	289.81	COOH	0.70	2
**MelCit P600**	284.64	C-C, C-H, C=C	11.33	23
	286.23	C-N, C-O	8.47	17
	288.13	C≡N-C	29.15	60
**MelCit P700**	284.58	C-C, C-H, C=C	20.90	37
	285.93	C-N, C-O	8.45	15
	287.21	C≡N-C, C=O	23.04	40
	288.86	COOH	4.46	8
**MelCit P750**	284.60	C-C, C-H, C=C	33.65	49
	285.77	C-N, C-O	16.53	24
	286.99	C=N, C=O	14.03	21
	288.84	COOH	4.22	6
**MelCit P800**	284.59	C-C, C-H, C=C	45.97	62
	286.05	C-N, C-O	21.05	28
	287.92	C=N, C=O	5.65	8
	289.61	COOH	1.88	2
**MelCit P850**	284.58	C-C, C-H, C=C	52.49	67
	285.98	C-N, C-O	20.07	26
	287.68	C=N, C=O	4.21	5
	288.84	COOH	1.33	2
**MelCit P900**	284.60	C-C, C-H, C=C	54.78	67
	285.81	C-N, C-O	14.83	18
	286.96	C=N, C=O	8.81	11
	288.96	COOH	3.13	4
**MelCit P950**	284.63	C-C, C-H, C=C	67.00	75
	285.83	C-N, C-O	9.93	11
	286.74	C=N, C=O	9.52	11
	288.71	COOH	2.57	3

Table S5. Relative percentages obtained from the deconvolution of the XPS high resolution N 1s spectra of MelCit 2:1 carbons obtained after pyrolysis at different temperatures.

Sample	Energy (eV)	Species	at. %	%
**MelCit P500**	398.78	C≡N-C	35.13	71
	400.40	Pyrrolic	11.05	23
	401.47	Quaternary	2.11	4
	404.38	N-oxide	0.97	2
**MelCit P600**	398.57	C≡N-C	29.02	65
	400.27	Pyrrolic	15.88	35
**MelCit P700**	398.31	Pyridinic	21.49	60
	400.09	Pyrrolic	14.42	40
**MelCit P750**	398.24	Pyridinic	13.23	52
	400.01	Pyrrolic	10.72	42
	401.23	Quaternary	1.50	6
**MelCit P800**	398.11	Pyridinic	9.25	50
	399.91	Pyrrolic	8.05	43
	401.26	Quaternary	1.25	7
**MelCit P850**	398.12	Pyridinic	8.21	53
	399.97	Pyrrolic	6.48	42
	401.52	Quaternary	0.72	5
**MelCit P900**	398.12	Pyridinic	5.19	49
	399.83	Pyrrolic	3.86	40
	401.14	Quaternary	1.17	11
**MelCit P950**	398.19	Pyridinic	2.83	48
	399.87	Pyrrolic	2.48	40
	401.3	Quaternary	0.87	12

Table S6. Conversion (%) vs time during the five cyclic experiments showing the reusability of Mel-Cit P850 carbon in the hydrogenation of 1-chloro-4-nitrobenzene.

Time (min)	Cycle 1	Cycle 2	Cycle 3	Cycle 4	Cycle 5
**0**	0.78	0.58	0.32	0.15	0.22
**15**	4.00	1.50	1.40	1.28	0.10
**30**	5.97	3.62	3.25	2.95	2.53
**60**	10.53	5.27	4.12	4.35	4.57
**120**	17.87	10.17	8.23	8.10	7.98
**180**	22.51	15.63	12.54	10.92	11.02
**300**	30.97	25.24	22.13	20.12	19.85
**360**	35.27	29.52	26.32	25.12	24.98
**480**	44.40	39.25	35.41	34.23	33.12
**600**	54.48	49.32	46.85	43.68	41.95
**900**	75.32	65.45	62.12	58.21	55.23
**1200**	92.60	82.05	77.85	69.45	65.21
**1440**	100.00	91.45	85.63	77.19	72.85
**1800**		100.00	90.23	82.35	80.12

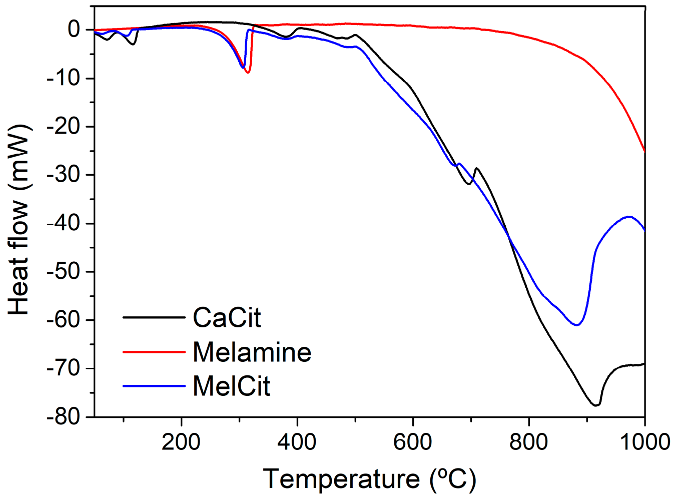

Figure S1. DSC profiles of calcium citrate tetrahydrate, melamine, and the corresponding physical mixture of both (2:1 w/w ratio). Heating rate: 5 ºC/min; N_2 atmosphere.

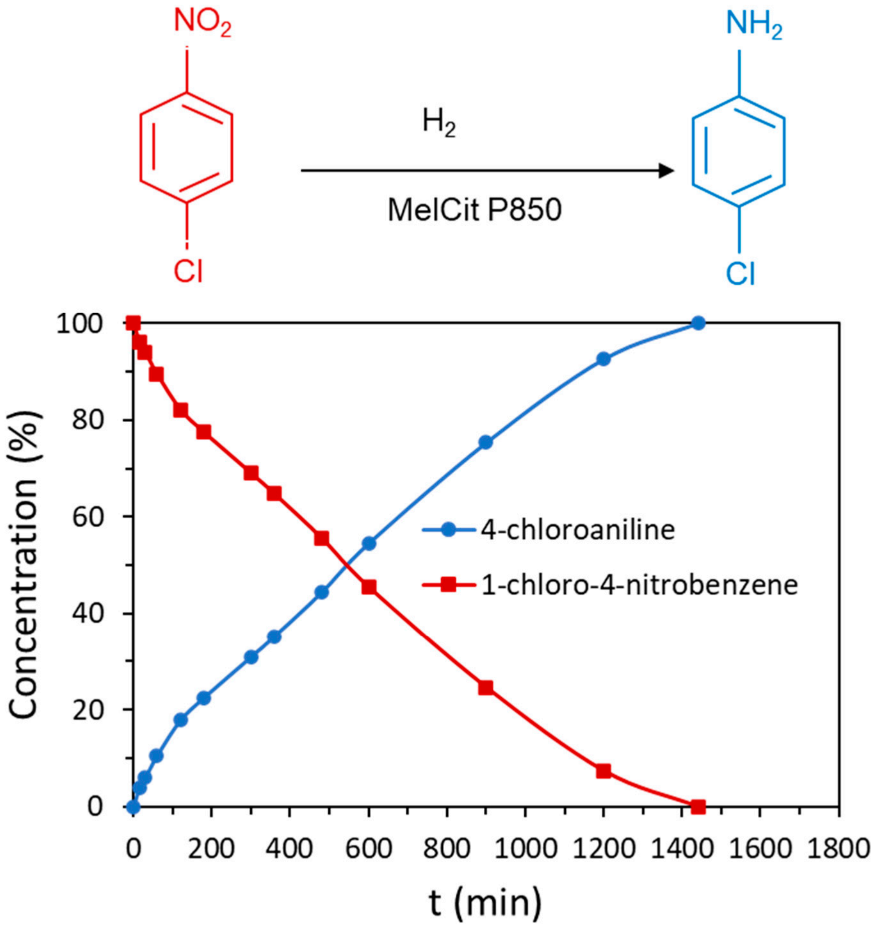

Figure S2. Evolution of compounds over the course of the hydrogenation reaction in the presence of MelCit P850 carbon.

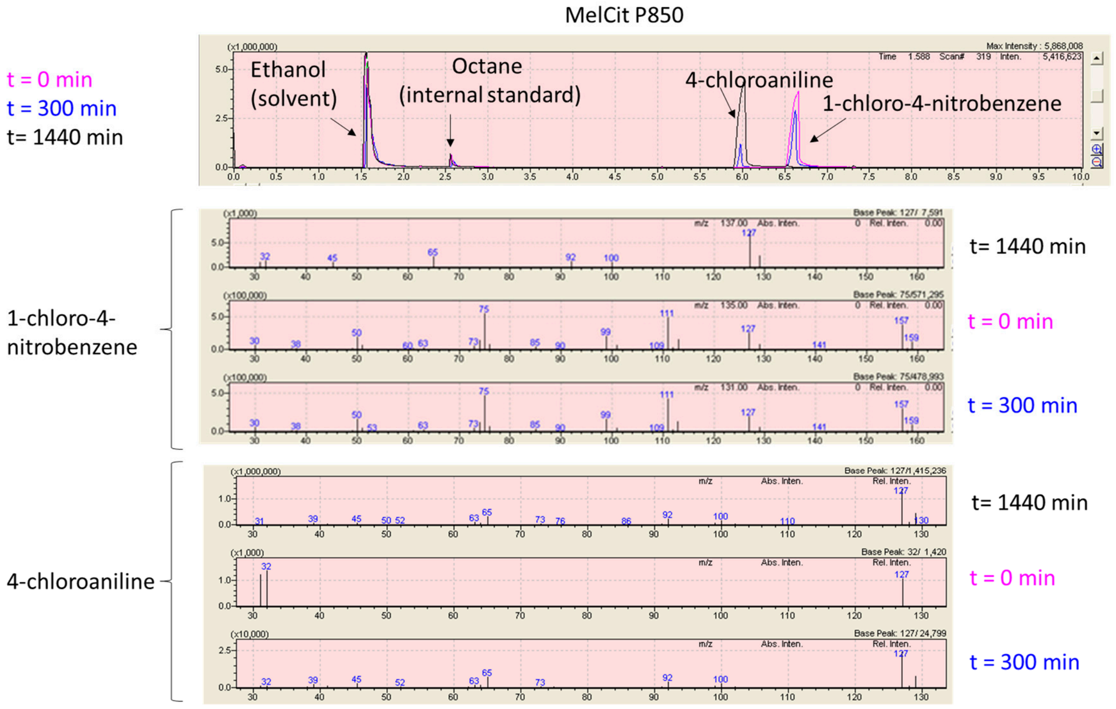

Figure S3. GC-MS analysis of compounds at t = 0 min, t= 300 min and t= 1440 min during the course of the hydrogenation reaction in the presence of MelCit P850 carbon.

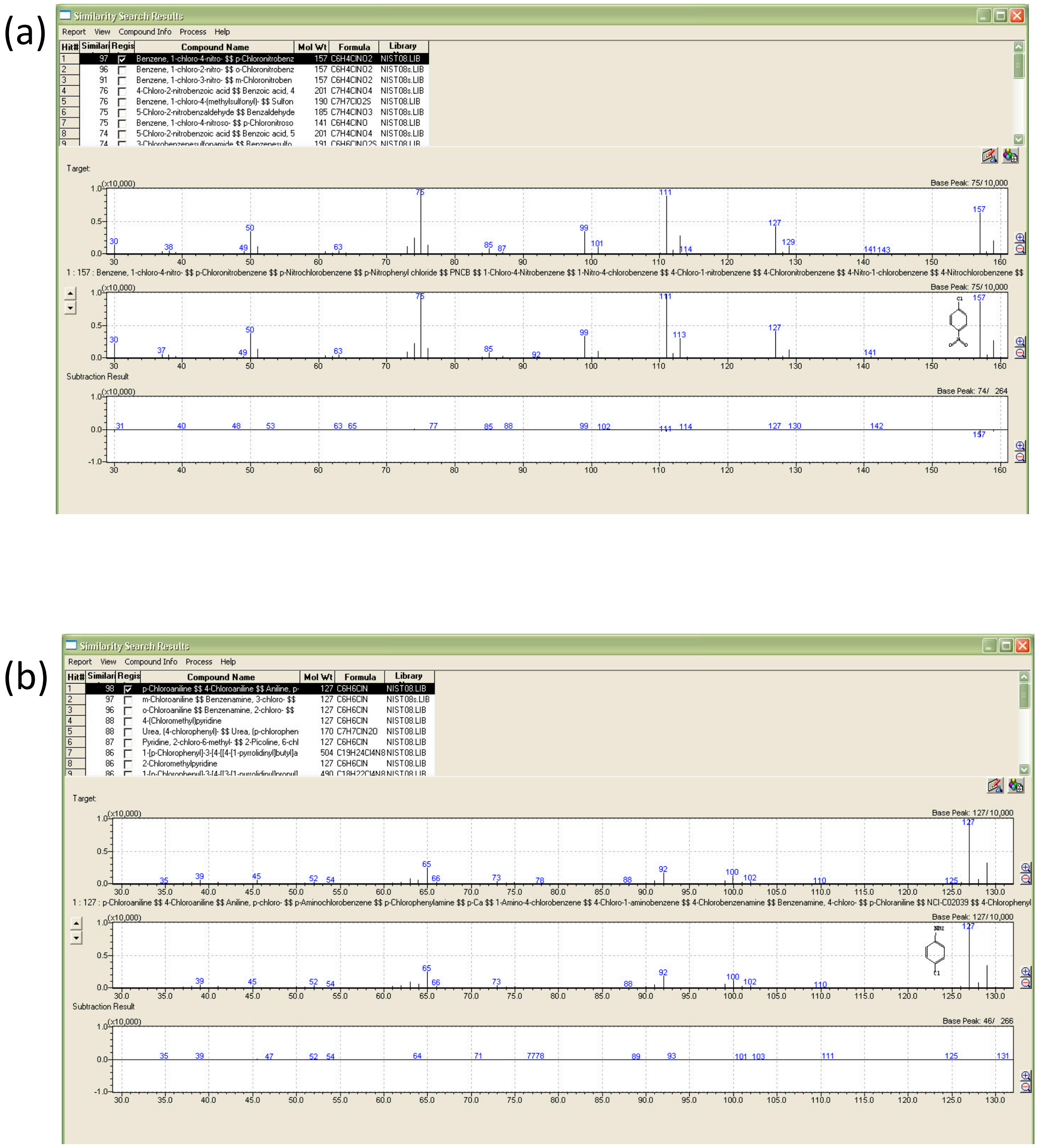
(a)
Similarity Search Results
Report View Compound Info Process Help
Hit# Similar Regis Compound Name Mol Wt Formula Library
1 97 Benzene, 1-chloro-4-nitro- $$ p-Chloronitrobenz 157 C6H4ClNO2 NIST08.LIB
2 96 Benzene, 1-chloro-2-nitro- $$ o-Chloronitrobenz 157 C6H4ClNO2 NIST08s.LIB
3 91 Benzene, 1-chloro-3-nitro- $$ m-Chloronitroben 157 C6H4ClNO2 NIST08s.LIB
4 76 4-Chloro-2-nitrobenzoic acid $$ Benzoic acid, 4 201 C7H4ClNO4 NIST08s.LIB
5 76 Benzene, 1-chloro-4-(methylsulfonyl)- $$ Sulfon 190 C7H7ClO2S NIST08.LIB
6 75 5-Chloro-2-nitrobenzaldehyde $$ Benzaldehyde 185 C7H4ClNO3 NIST08s.LIB
7 75 Benzene, 1-chloro-4-nitroso- $$ p-Chloronitroso 141 C6H4ClNO NIST08.LIB
8 74 5-Chloro-2-nitrobenzoic acid $$ Benzoic acid, 5 201 C7H4ClNO4 NIST08s.LIB
Target:
Base Peak: 75/ 10,000
1 : 157 : Benzene, 1-chloro-4-nitro- $$ p-Chloronitrobenzene $$ p-Nitrochlorobenzene $$ p-Nitrophenyl chloride $$ PNCB $$ 1-Chloro-4-Nitrobenzene $$ 1-Nitro-4-chlorobenzene $$ 4-Chloro-1-nitrobenzene $$ 4-Chloronitrobenzene $$ 4-Nitro-1-chlorobenzene $$ 4-Nitrochlorobenzene $$
Subtraction Result
Base Peak: 74/ 264
(b)
Similarity Search Results
Report View Compound Info Process Help
Hit# Similar Regis Compound Name Mol Wt Formula Library
1 98 p-Chloroaniline $$ 4-Chloroaniline $$ Aniline, p- 127 C6H6ClN NIST08.LIB
2 97 m-Chloroaniline $$ Benzenamine, 3-chloro- $$ 127 C6H6ClN NIST08s.LIB
3 96 o-Chloroaniline $$ Benzenamine, 2-chloro- $$ 127 C6H6ClN NIST08.LIB
4 88 4-(Chloromethyl)pyridine 127 C6H6ClN NIST08.LIB
5 88 Urea, (4-chlorophenyl)- $$ Urea, (p-chlorophen 170 C7H7ClN2O NIST08.LIB
6 87 Pyridine, 2-chloro-6-methyl- $$ 2-Picoline, 6-chl 127 C6H6ClN NIST08.LIB
7 86 1-[p-Chlorophenyl]-3-[4-[[4-[1-pyrrolidinyl]butyl]a 504 C19H24Cl4N8 NIST08.LIB
8 86 2-Chloromethylpyridine 127 C6H6ClN NIST08.LIB
Target:
Base Peak: 127/ 10,000
1 : 127 : p-Chloroaniline $$ 4-Chloroaniline $$ Aniline, p-chloro- $$ p-Aminochlorobenzene $$ p-Chlorophenylamine $$ p-Ca $$ 1-Amino-4-chlorobenzene $$ 4-Chloro-1-aminobenzene $$ 4-Chlorobenzenamine $$ Benzenamine, 4-chloro- $$ p-Chloraniline $$ NCI-C02039 $$ 4-Chlorophenyl
Subtraction Result
Base Peak: 46/ 266

(c)

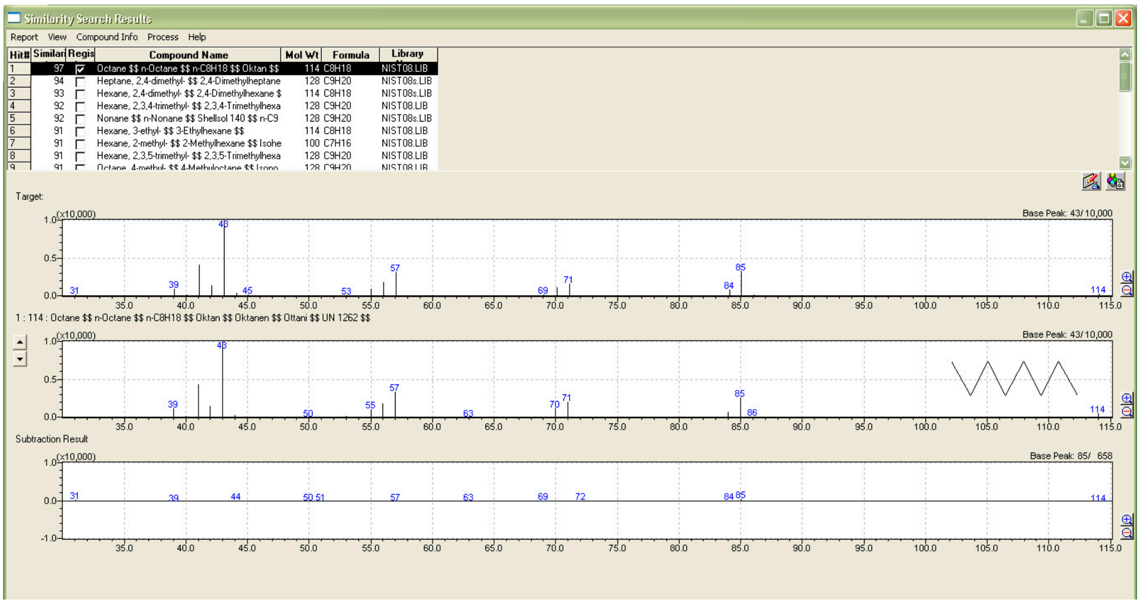

(d)

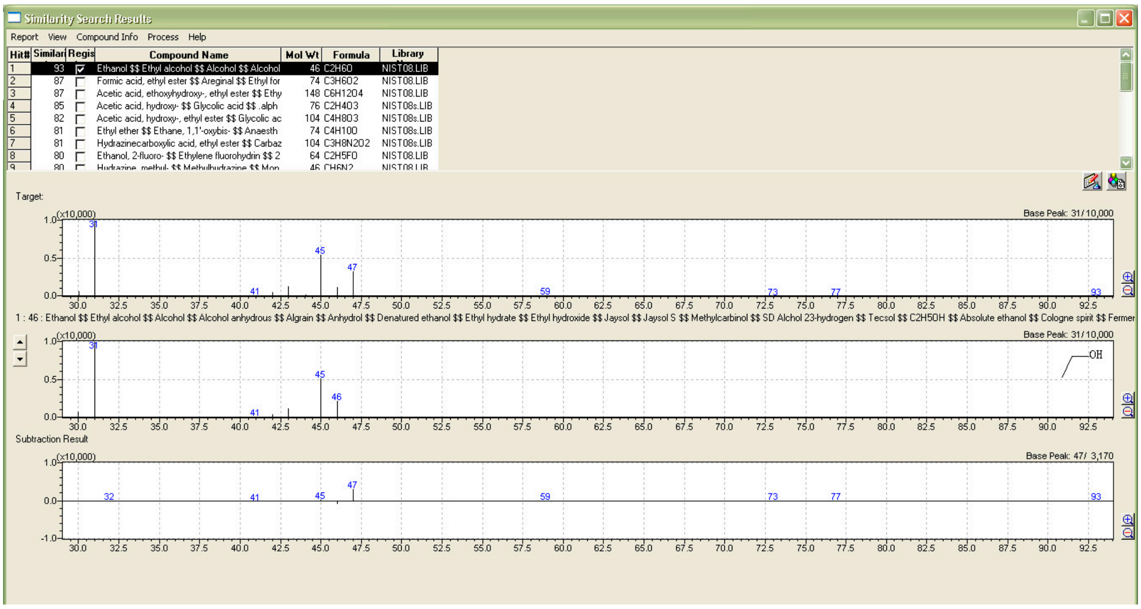

Figure S4. Mass spectra (MS) similarity search results of compounds detected during the course of the hydrogenation reaction in the presence of MelCit P850 carbon showing (a) 1-chloro-4-nitrobenzene, (b) 4-chloroaniline, (c) octane (internal standard) and (d) ethanol (solvent).
